# Synthesis and cytotoxic activity evaluation of novel imidazopyridine carbohydrazide derivatives

**DOI:** 10.1186/s13065-023-01073-3

**Published:** 2024-01-06

**Authors:** Maryam Firouzi, Zahra Haghighijoo, Masoomeh Eskandari, Maryam Mohabbati, Ramin Miri, Mohammad Hasan Jamei, Alireza Poustforoosh, Somayeh Nazari, Omidreza Firuzi, Mehdi Khoshneviszadeh, Najmeh Edraki

**Affiliations:** 1 Medicinal and Natural Products Chemistry Research Center, Shiraz University of Medical Sciences, Shiraz, Iran; 2 Department of Medicinal Chemistry, Faculty of Pharmacy, Shiraz University of Medical Sciences, Shiraz, Iran

**Keywords:** Imidazopyridine, 1,2,3-triazole ring, Carbohydrazide, Cytotoxic activity, Molecular docking, Molecular dynamics

## Abstract

Two series of novel imidazo[1,2-a]pyridine-2-carbohydrazide derivatives have been designed, synthesized, and evaluated for cytotoxic activity. Target compounds were designed in two series: aryl hydrazone derivatives that were devoid of triazole moiety (7a-e) and aryl triazole bearing group (11a-e). In vitro cytotoxicity screening was carried out using MTT assay against three human cancer cells including breast cancer (MCF-7), colon cancer (HT-29), and leukemia (K562) cell lines as well as a non-cancer cell line (Vero). Compound 7d bearing 4-bromophenyl pendant from aryl hydrazone series exhibited the highest cytotoxic potential with IC_50_ values of 22.6 µM and 13.4 µM against MCF-7 and HT-29 cells, respectively, while it was not toxic towards non-cancer cells up to the concentration of 100 µM. Cell cycle analysis revealed that 7d increased the number of MCF-7 cells in the G0/G1 phase and also induced apoptosis in these cells as revealed by Hoechst 33,258 staining. The molecular mechanism contributing to the anti-proliferative effect of the most potent compound was investigated in silico using Super Pred software and introduced PDGFRA as a plausible target for 7d. Molecular docking and molecular dynamic studies demonstrated Lys627 and Asp836 as key residues interacting with the active compound. Overall, 7d could serve as a suitable candidate for further modifications as a lead anticancer structure.

**Supplementary Information:**

The online version contains supplementary material available at 10.1186/s13065-023-01073-3.

## Introduction

Despite considerable recent advances in the treatment of cancer, the mortality rate of this disease remains very high worldwide and is still a major health concern. According to the GLOBOCAN 2020 database, the number of new cancer cases is estimated roughly 19.3 million patients in 2020. In this regard, one of the world’s top medical priorities should be the development of new therapeutic options for this deadly disease [1].

**Fig. 1:**
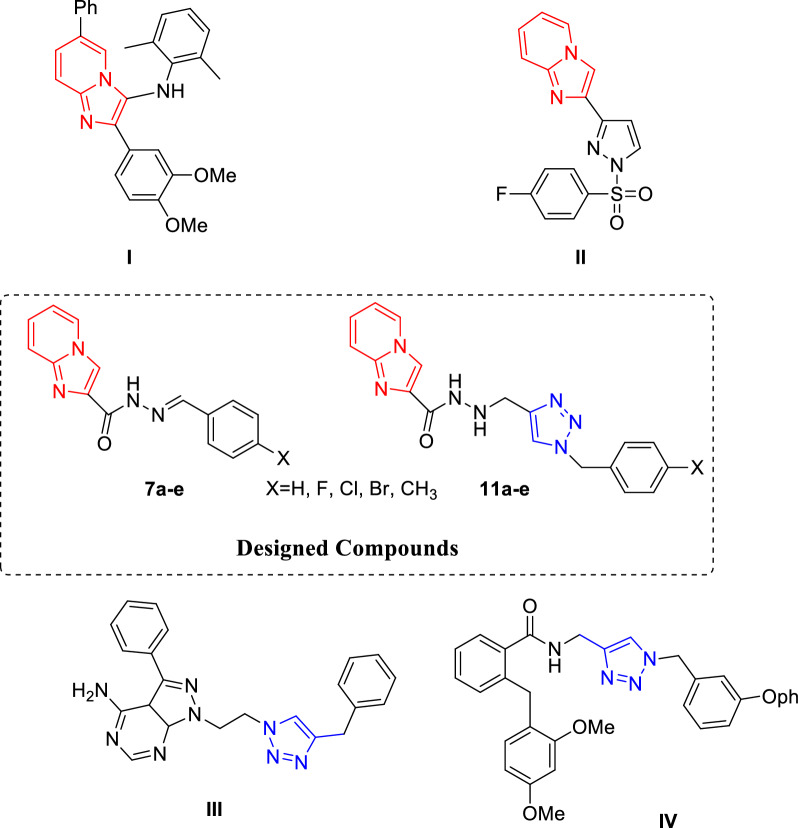
Chemical structure of some anti-cancer agents containing imidazo [1,2-a] pyridine and 1,2,3-triazol

The imidazopyridine scaffold possesses a broad range of activities including anxiolytic, antiulcer, anti-mycobacterial, antiviral, anti-inflammatory, and anticancer properties [2–5]. Recently, during the investigation of imidazopyridine derivatives with anticancer properties, some 6-substituted imidazo[1,2-a] pyridine derivatives have been found to be effective against colon cancer cell lines (compound I, Fig. 1) [6]. Another effort on structural modification of these series containing pyrazole moiety showed the potential of these molecules as potent and selective p110a inhibitors (compound II, Fig. 1) [7]. On the other hand, 1,2,3-triazole-based structures have been previously reported as anti-cancer agents (Compound III, Fig. 1) [8–13]; Compound IV from triazole-linked nicotinamides exhibited promising antiproliferative activity and ability to inhibit tubulin polymerization. In this regard, the triazole linkage would presumably be favorable for potent and selective anticancer agents [14–16].

Platelet-derived growth factor receptor alpha (PDGFRA) is a protein tyrosine kinase that can activate some pathways, such as Raf/MEK/ERK and RAS/MAPK [17]. These pathways can induce cell proliferation, angiogenesis, and tumor cell growth. Previous studies have reported the essential role of PDGFRA in various cancers, such as breast cancer, colorectal cancer, and leukemia [18–20].

As a continuation of our interest in the design of novel anticancer agents [21–24], herein we report the design and synthesis of imidazopyridine carbohydrazide derivatives characterized by an imidazopyridine core and two different substituted pendants. This strategy yielded two different series: aryl hydrazone derivatives lacking triazole moiety **(7a-e)** and aryl triazole group **(11a-e)**. Various aryl moieties were subjected to gradual structural alteration to investigate the importance of their contribution to the enhancement of cytotoxic potency. Afterwards, the synthesized compounds were evaluated for their cytotoxic potential against three human cancer cell lines MCF-7, HT-29, and K562 cells. Furthermore, the distribution of cancer cells incubated with the most potent compound 7d in different phases of the cell cycle was studied in order to understand the mode of action of these compounds (Additional file 1).

## Results and discussion

### Chemistry

**Fig. 2:**
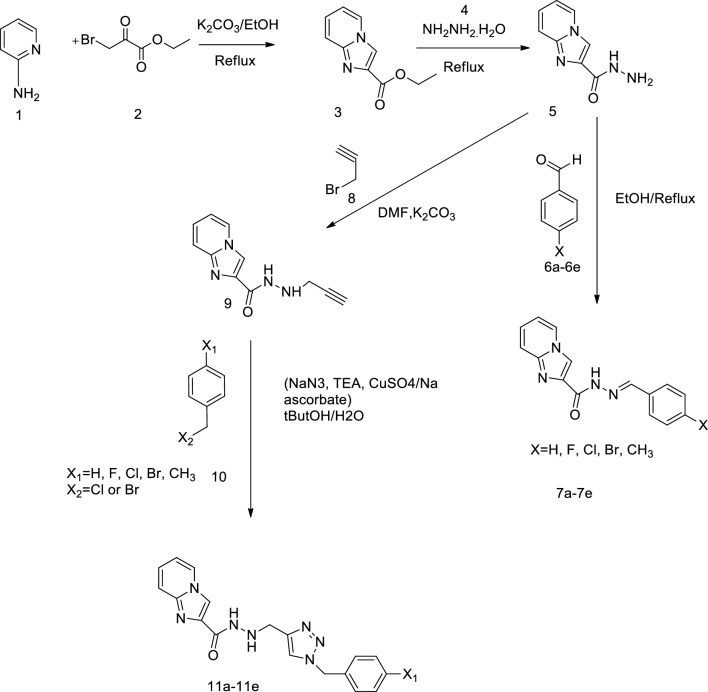
The synthesis procedure of target compounds (**7a-e**, **11a-e**)

A series of novel imidazo[1,2-a]pyridine-2-carbohydrazide derivatives (**7a-e**, **11a-e**) were synthesized in several steps as outlined in Fig. 2. The chemical reaction of aminopyridine **1** and ethyl 3-bromo-2-oxo propanoate **2** in refluxing ethanol led to the synthesis of ethylimidazo[1,2-a]pyridine-2-carboxylate **3**. In the second step, treatment of ester intermediate **3** with an excess amount of hydrazine hydrate afforded carbohydrazide derivative **5** under a refluxing condition. ^1^HNMR spectrum of **5** represented two singlet peaks at 9.53 and 4.48 ppm which were attributed to a hydrazine group at position 2. In addition, the IR spectrum of **5** showed the appearance of the band at 1660 cm, the characteristic of carboxamide group stretch. Different substituted aldehydes **6a-e** were reacted with intermediate **5** to afford the corresponding benzylidene imidazo[1,2-a]pyridine-2-carbohydrazides **7a-e** [25]. In the ^1^HNMR spectrum of **7a-e**, the disappearance of the proton shifts of NH_2_ and the presence of the singlet peaks (related to the protons N = CH) were obtained as the result of the further formation of the imine bond in the corresponding products. Further reaction of **5** with propargyl bromide in the presence of K_2_CO_3_ in refluxing DMF resulted in the corresponding prop-2-yn-1-yl imidazo[1,2-a]pyridine-2-carbohydrazide **9**. ^1^HNMR spectrum of **9** presented two singlet peaks at 3.70 and 2.23 ppm which corresponded to a 2-propynyl group. The click reaction of **9** with different in situ*-*produced azides, catalyzed by CuSO_4_.5H_2_O in the presence of sodium ascorbate afforded the final product **11a-e**. The ^1^HNMR spectrums were consistent with the assigned structures of triazole, benzyl-substituted pendant, and all other aromatic protons at expected regions.

### Biological activity

The results of in vitro cytotoxic activity of imidazopyridine derivatives are summarized in Table 1. All synthesized compounds were evaluated for cytotoxic activity against three human cancer cell lines including MCF-7, HT-29, and K562 by employing an MTT (3-(4,5-dimethylthiazol-2-yl)-2,5-diphenyltetrazolium bromide) assay. The most active derivatives were also examined against Vero non-cancer cells. Cisplatin and doxorubicin were tested as standard chemotherapeutic reference agents.

**Table 1:** Cytotoxic effects of synthesized compounds against MCF-7, HT-29 and K562 cancer cell lines and vero non-cancer cells examined by MTT reduction assay expressed as IC_50_ values (µM)

Compound		MCF-7^A^	HT-29	K562	Vero	miLog P^B^
**5**		> 100^C^	> 100	> 100	–^D^	−0.08
**9**		70.1 ± 5.3	21.4 ± 1.9	23.7 ± 2.3	73.0 ± 9.9	0.65
**7a**		> 100	> 100	> 100	–	2.70
**7b**		> 100	> 100	> 100	–	2.86
**7c**		64.8 ± 11.8	85.4 ± 10.3	> 100	65.6 ± 4.5	3.38
**7d**		22.6 ± 1.0	13.4 ± 1.3	> 100	> 100	3.51
**7e**		> 100	> 100	> 100	–	3.15
**11a**		40.3 ± 2.9	61.2 ± 6.3	> 100	> 100	1.64
**11b**		> 100	> 100	> 100	–	1.80
**11c**		> 100	> 100	> 100	–	2.32
**11d**		> 100	83.8 ± 13.1	> 100	> 100	2.00
**11e**		> 100	> 100	> 100	–	2.09
Cisplatin	–	25.9 ± 6.9	22.4 ± 5.8	9.4 ± 0.5	10.6 ± 1.3	
Doxorubicin		329.7^E^ ± 130.8	750.3^E^ ± 123.1	44.9^E^ ± 2.4	2.6 ± 0.3	

^A^IC_50_ values against each cell line are expressed in µM ± S.E.M

^B^log P was measured by an online tool at http://www.molinspiration.com/cgi-bin/properties website

^C^The cytotoxic effect did not reach 50% at the maximum tested concentration of 100 µM

^D^Not tested

^E^Data expressed in nM

Overall, it was observed that imidazopyridine derivatives (**7a-7e**) exhibited superior cytotoxicity compared to imidazopyridine triazole counterparts (**11a-11e**) against tested cell lines. Five compounds (**9**, **7c**, **7d**, **11a,** and **11d**) exhibited weak to moderate cytotoxic activity with IC_50_ values as low as 22.6 μM (MCF-7), 13.4 μM (HT-29) and 23.7 μM (K562). None of the compounds except derivative **9** showed any cytotoxicity against K562 cell lines up to the highest tested concentration of 100 µM. Among all evaluated derivatives, compound **7d** containing bromide substitution showed the best cytotoxic activity with IC_50_ values of 22.6 and 13.4 µM against MCF-7 and HT-29 cell lines, respectively. However, the introduction of triazole linkage between substituted phenyl and imidazopyridine in the other bromide-bearing compound, **11d,** decreased the activity against the HT-29 cell line with the IC_50_ values of 83.9 µM and no activity was observed in the two other cell lines.

Assessment of the cytotoxic activity and preliminary structure–activity relationship (SAR) of tested compounds revealed that lipophilicity might be an effective parameter. So a mild change in activity was found between the intermediate of the template structure of imidazopyridine-2-carbohydrazide **5** and propyn-1yl imidazopyridine-2-carbohydrazide** 9** with an increase in logP value from −0.08 to 0.65. Considering lower cytotoxic activity and logP of compound **7c** bearing chlorine substitute (IC_50_ values of 64.8 and 85.4 µM against MCF-7 and HT-29 cell lines, respectively) compared to **7d**, it could be implied that the lipophilicity parameter has an indispensable role on the cytotoxic potential of these series of compounds. A linear dependency between lipophilicity and cytotoxicity could be observed so that the more lipophilic compounds might have improved cytotoxic effects.

On the other hand, in vitro screening results of the triazole-bearing analogs revealed that the compound **11a** containing benzyl moiety with a logP value of 1.64 demonstrated superior cytotoxic activity against both MCF-7 and HT-29 cells compared to **11d** with a logP value of 2.0. Hence, it could be construed that in the **11a-11e** compounds, unlike the **7a-7e** series, higher lipophilicity is not necessarily associated with improved cytotoxicity.

As can be noted in Table 1, the compounds **7d**, **11a,** and **11d** were completely inactive against Vero non-cancer cells, while derivative **9** showed an IC_50_ value of 73.0 µM, which was higher than the IC_50_ values observed against cancer cells. Only compound **7c** showed an IC_50_ value of 65.6 µM, which was comparable with the effect observed against cancer cells.

**Fig. 3:**
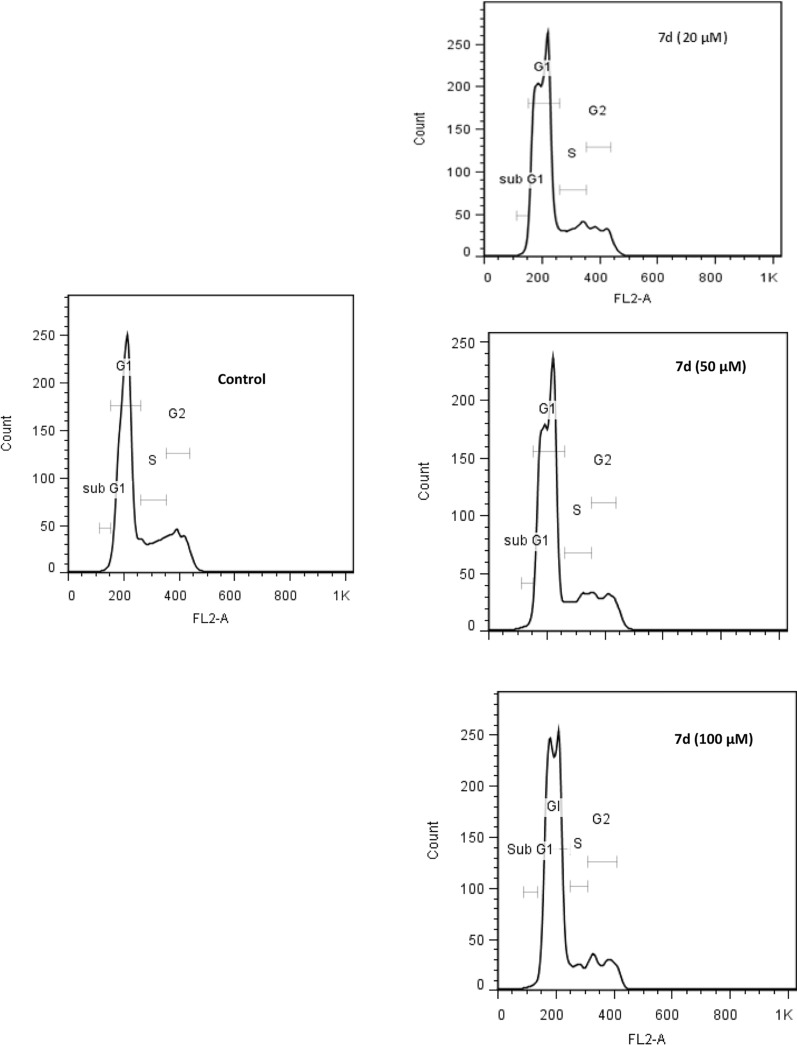
Effect of compound **7d** on cell cycle phase distribution of MCF-7 cells. The analysis of cells in different phases of the cell cycle was performed using propidium iodide (PI)-based assessment of cell cycle by flow cytometry. MCF-7 cells were seeded in 12-well plates and treated with different concentrations of **7d** (20, 50, and 100 nM) for 48 h. The cells were then collected, washed with PBS and fixed with 70% ethanol overnight at −20 °C. After 24 h, the fixed cells were incubated in a PI-RNase solution at room temperature and 10,000 cells were analyzed using a FACS Calibur flow cytometer (BD Biosciences)

Furthermore, the arrest of cancer cells at any phase of the cell cycle caused by certain anti-cancer agents prevents proliferation and could be exploited as a therapeutic strategy. In order to explore the possible mechanisms of action of the most potent imidazopyridine derivative, the cell-cycle distribution of MCF-7 cells incubated with **7d** was evaluated using propidium iodide (PI)-based assessment of cell cycle by flow cytometry. The distribution of treated and control cells in different phases of the cell cycle is presented in Table 2 and Fig. 3. The obtained data clearly indicated that this compound caused a dose-dependent increase in the G0/G1 phase and a concomitant decrease in the G2/M phase. These data indicate that the compounds cause a prolongation or arrest in the G1 phase or probably increase the number of cells that leave the cycle and enter the G0 phase.

**Table 2:** Percent distribution of MCF-7 cells in phases of cell cycle in the presence or absence of **7d**

Sample	Sub G1	G0/G1	S	G2/M
Control	0.8 ± 0.3	61.4 ± 1.4	11.7 ± 2.3	26.1 ± 2.9
7d (20 µM)	0.8 ± 0.6	64.2 ± 7.6	11.9 ± 5.7	23.3 ± 10.0
7d (50 µM)	0.8 ± 0.6	67.0 ± 8.4	11.1 ± 4.9	21.1 ± 8.8
7d (100 µM)	1.1 ± 0.9	67.2 ± 7.5	10.2 ± 5.3	21.5 ± 7.2

**Fig. 4:**
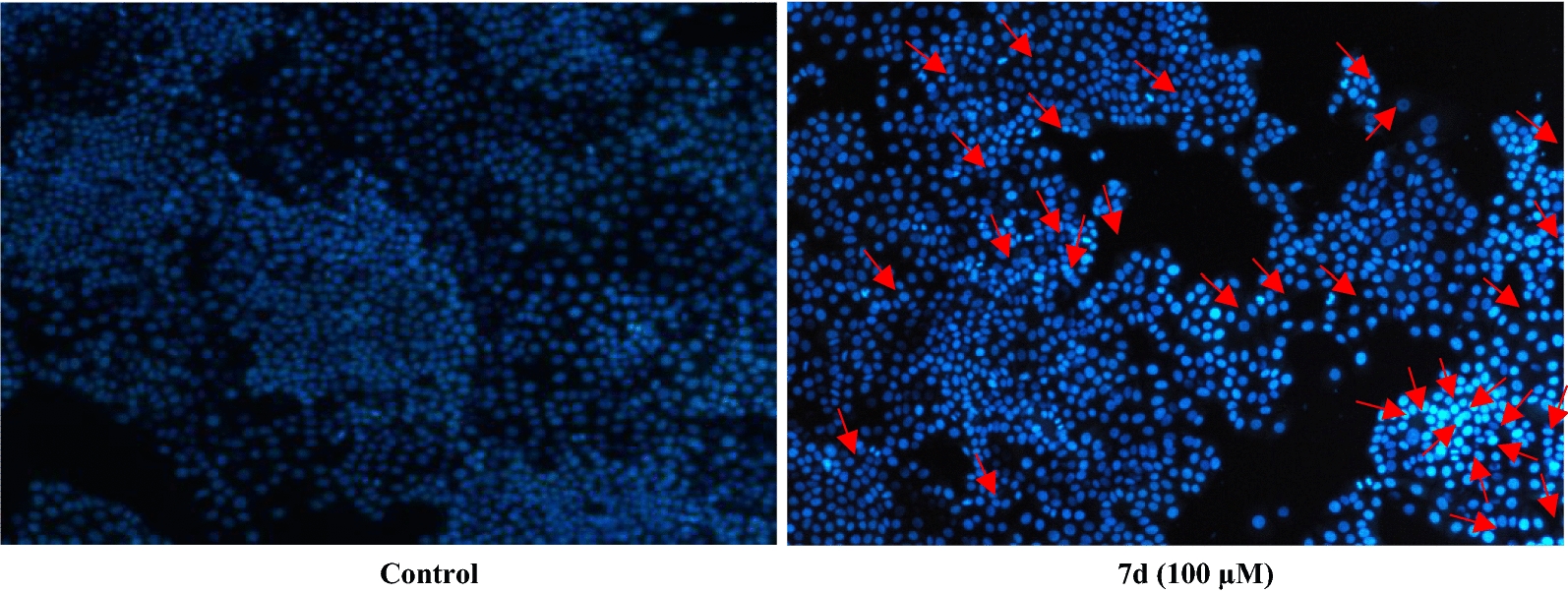
Measurement of apoptosis induced by Hoechst 33,258 staining assay. MCF-7 cells were cultured in 6-well plates at a density of 10^5^ cells/ml and exposed to 100 µM of the most potent compound (**7d**) for 72 h. The cells were then stained with 2.5 µg/ml Hoechst 33,258 after being fixed with 4% cold paraformaldehyde (PFA) and imaged using a fluorescence microscope (Magnification: 500 X). Apoptotic cells as featured by nuclear shrinkage, fragmentation, condensation, etc., are shown by red arrows

In addition, we also observed that the most active compound, **7d**, was able to induce apoptosis in MCF-7 cells as revealed by the Hoechst 33,258 staining assay (Fig. 4). This compound showed clear signs of apoptosis including nuclear condensation and fragmentation at the concentration of 100 µM. This also further adds to the potential value of this derivative as an anticancer agent, as it is known that apoptosis evasion is an important hallmark of cancer, and apoptosis induction capacity is an important feature of any successful therapeutic agent.

### Computational studies

#### Target prediction

Considering the promising cytotoxic potential of some synthesized compounds, a computational approach was performed to find some insights about the probable target of designed scaffolds. Due to the obtained results from SuperPred target prediction and the CGC database, PDGFRA was selected to be further evaluated by molecular docking calculations. SuperPred target prediction online tool was used to find the most probable targets. This tool uses machine learning models emphasizing the significance of functional groups for the mechanism of action of small molecule substances. Our previous research study on imidazopyridines also revealed PDGFRA as a potential target of this backbone [26]. In another study accomplished by Sato et al. imidazopyridines were designed to inhibit SIK2 and SIK3, and the off-target assessment of derivatives indicated that PDGFRA is the major off-target with IC_50_ = 15.8 nM [27]. In addition, imidazopyridine carbohydrazide derivatives have been reported to have kinase inhibitory activity and can be used as potent inhibitors against protein tyrosine kinases [28].

#### Molecular docking 

**Fig. 5:**
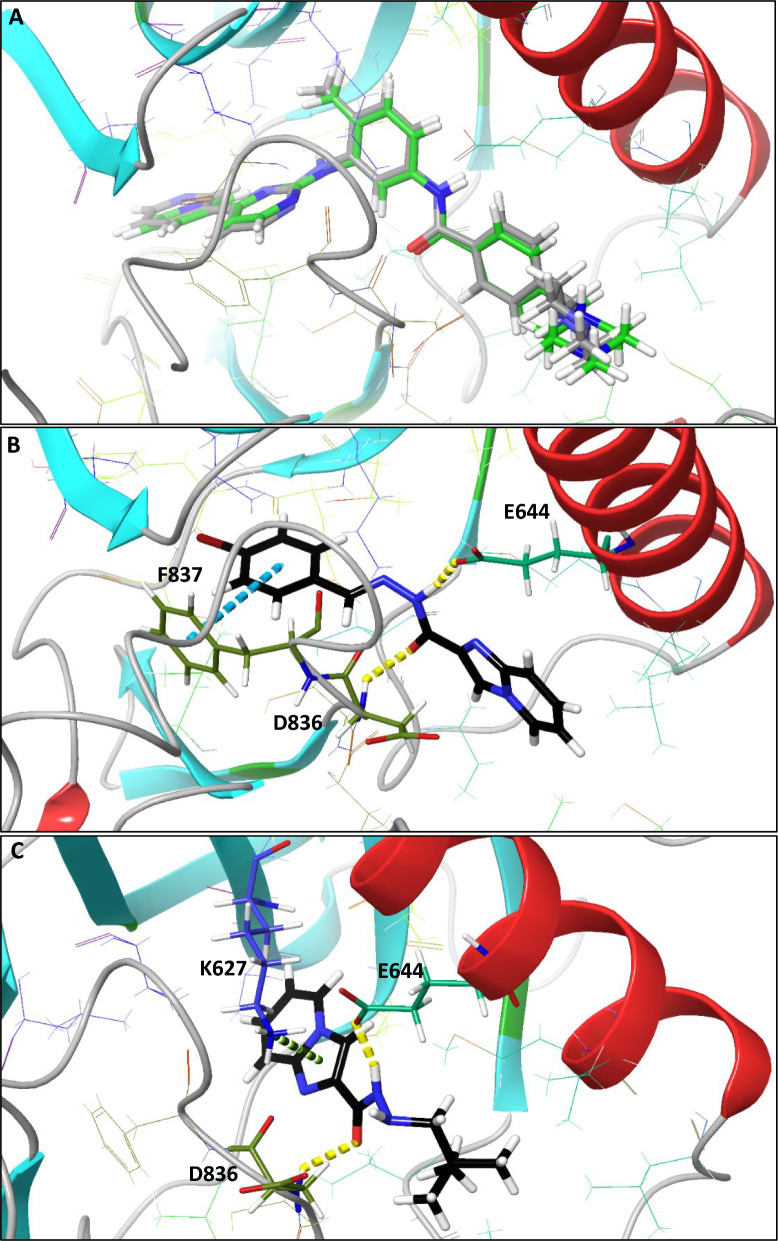
**A**: Superimposition of imatinib docked into the drug-substrate binding site (green) and imatinib in the PDGFRA PDB (grey). **B**: The docking pose of **7d** in the active binding site of PDGFRA. **C**: The docking pose of **9** in the active binding site of PDGFRA (dashed lines: Yellow: H-bond, Blue: Pi-Pi stacking, Green: Pi-cation)

We selected PDGFRA as the plausible target of designed compounds to be further evaluated by molecular docking calculations. The structure used in this study was PDGFRA (PDB ID: 6JOL) from the PDB database (http://www.rcsb.org/pdb). The chemical activities of the synthesized compounds when interacting with PDGFRA were evaluated using molecular docking. The outcomes showed the binding affinity of the compounds to the targets and the probable residues of the protein that can create strong interactions with the compounds. The results of molecular docking calculations are presented in Table 3. Validation of molecular docking resulted in a root mean square deviation (RMSD) of 1.16 Å between the best pose of co-crystallized ligand (imatinib) docked into the drug-substrate binding site of PDGFRA and the one in the PDB (Fig. 5A). In general, the docking scores have a positive correlation with the experimental results, and compounds **7d** and **9** showed superior binding affinities in the PDGFRA active site. The docking pose of the compounds with the highest anti-cancer activity (**7d** and **9**) are shown in Fig. 5B, C. The interactions constructed between these compounds and PDGFRA are presented in Table 3. The amide moiety of compound **7d** has created two hydrogen bonds with Glu644 and Asp836 of PDGFRA. Glu644 and Asp836 are important residues for targeting this protein. Wang et al. have reported that their chemical compounds could create H-bond with these residues for inhibiting PDGFRA [29]. There is also a Pi-Pi stacking interaction between **7d** and Phe837. Phe837 previously has been reported by Wu et al. as an important residue for targeting PDGFRA [30]. There are also eleven hydrophobic contacts between the **7d** compound and PDGFRA, which can increase the binding affinity of the ligand to the protein. Compound **9** interacted via two hydrogen bonds with the residues Glu644 and Asp836 and a Pi-cation interaction with Lys627. The H-bonds are constructed by the amide moiety of the compound. The details of these interactions are presented in Table 3.

**Table 3:** The docking scores and details of interactions between the compounds and PDGFRA obtained from the molecular docking calculations

Compounds	Docking score (kcal/mol)	Interactions with PDGFRA	Residue	Length
7d	−7.584	H-bond H-bond Pi-Pi stacking	Glu644 Asp836 Phe837	1.81 Å 2.38 Å 5.38 Å
9	−7.137	Pi-cation H-bond H-bond	Lys627 Glu644 Asp836	3.84 Å 1.72 Å 2.16 Å

#### MD simulation

**Fig. 6:**
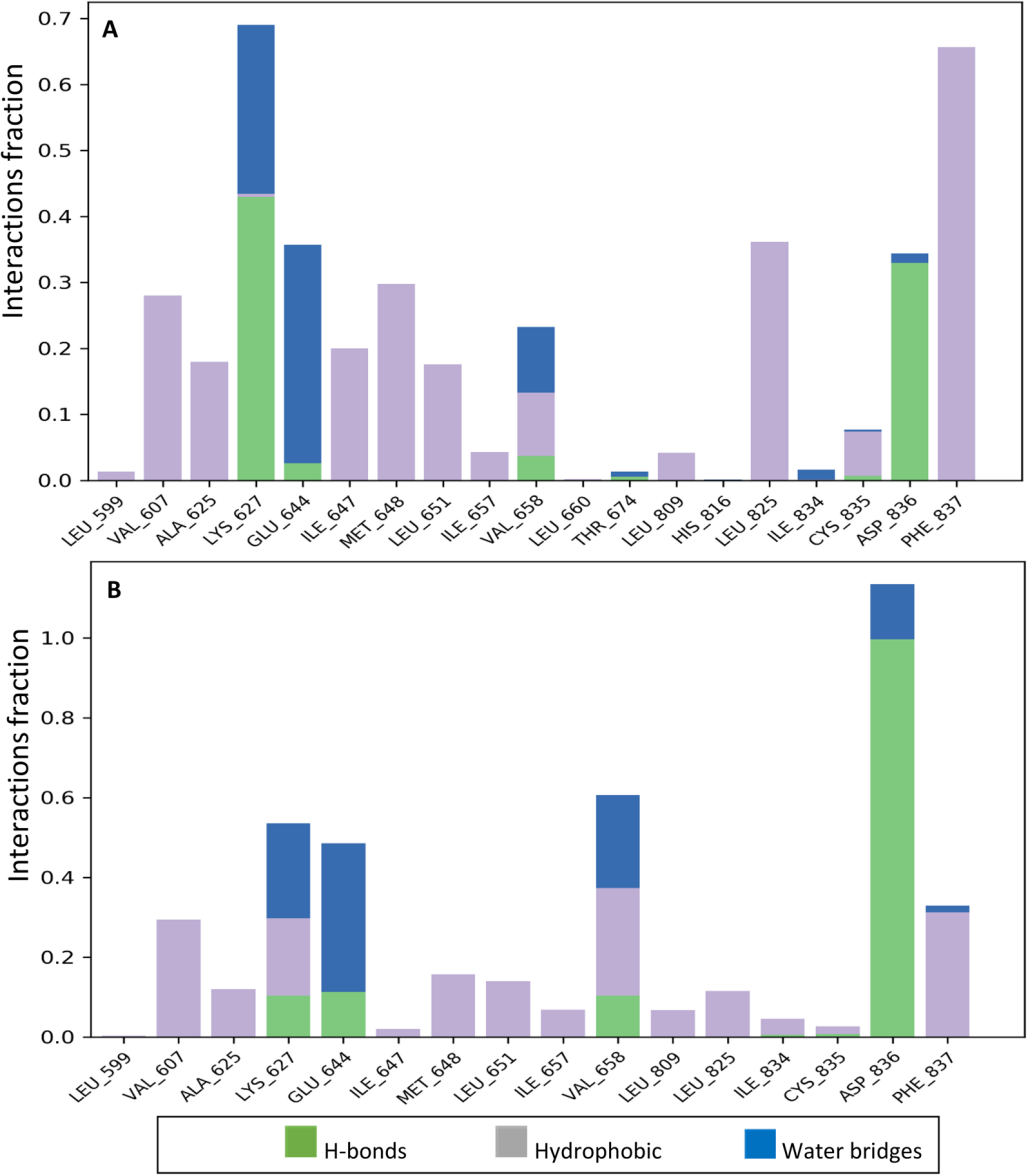
The interactions constructed between PDGFRA and the compounds** 7d** (**A**) and **9** (**B**) during the MD simulation

The ligand–protein complexes of promising compounds **7d** and **9** were further evaluated using the MD simulation for 100 ns. The RMSD value of the protein in the simulations of these compounds converged at about 1.8 Å indicating the stability of the systems after 100 ns. The ligand–protein interactions between PDGFRA and **7d** and **9** after the simulation time are presented in Fig. 6. Considering the results obtained for compound **7d**, the residues with the highest interaction fractions are Lys627 and Phe837. Although Lys627 did not show significant interaction in the docking study, some notable interactions are constructed in dynamic situations between Lys627 and **7d**. These two residues are essential for targeting this protein, which has been previously reported to construct some interactions such as H-bond with the PDGFRA inhibitors [30]. Whereas the major interactions constructed by Lys627 during the simulation are H-bond and water bridge, the major interaction for Phe837 is hydrophobic contact. The main target residue for **9** is H-bond interaction with Asp836. As indicated before, Asp836 has been reported as a key residue for the inhibition of PDGFRA [29].

## Conclusion

Some novel imidazopyridine derivatives have been designed and synthesized in two different groups: compounds bearing aryl hydrazine pendant (**7a-7e**) and aryl-triazole hydrazine bearing derivatives (**11a-11e**). We also investigated the role of 1,2,3-triazole linkage on the cytotoxic potential of the imidazopyridine derivatives against MCF-7, K562, and HT-29 cancer cells as well as Vero non-cancer cells. The results demonstrated that the triazole motif could not improve the cytotoxic potency of the tested compounds against any of the tested cell lines. Among them, compound **7d** from the imine series with the *para*-bromine substitution was found to be the most potent analog with IC_50_ values of 22.6 and 13.4 µM against MCF-7 and HT-29 cell lines, respectively, while it had no toxic effect against non-cancer cells. In addition, among triazole-bearing derivatives, compound **11a** showed moderate activity at IC_50_ values of 40.3 and 61.2 µM against the mentioned cell lines, respectively. The results indicated that the potent analog of this scaffold, **7d**, which could also increase the number of cancer cells in the G0/G1 phase of the cell cycle and induce apoptosis, may be considered as a suitable candidate for further modification as a lead anti-cancer structure.

Comparing the results of the present work with our previous studies clarifies the importance of aryl hydrazinyl pendant on the cytotoxic potential of studied backbones [22, 23]. Our previous studies on the 1,2,4-triazines and phenanthrotriazine derivatives bearing aryl-hydrazinyl pendant demonstrated the promising cytotoxic potential of some derivatives against cancer cell lines. Although the introduction of the 1,2,3-triazole linker between hydrazinyl group and aryl pendant diminishes the cytotoxic potential of target compounds, it might affect their selectivity towards the target, especially tyrosine kinases such as PDGFRA as evident in our previous work on imidazopyridine backbone [26]. In silico target prediction of the most potent compounds of this study using SuperPred software also introduced PDGFRA as a potential target of compound **7d**. Molecular docking and molecular dynamic studies demonstrated Lys627 and Asp836 as key binding interacting residues. Finally, some imidazopyridine hydrazinyl derivatives appear to be promising targeted compounds warranting further investigation as anticancer agents. Future studies on the PDGFRA and other kinases will be conducted to confirm molecular targets involved in the anticancer activity of the designed backbone. The present study would be the starting point for further studies and modification of anti-cancer imidazopyridines.

## Experimental methods

### Materials

All reagents and solvents were obtained from commercial suppliers and were used without further purification. The progress of the reactions was checked by thin-layer chromatography (TLC) using aluminum sheets coated with silica gel 60-F254 (0.5 mm) (Merck, Darmstadt, Germany).

### Apparatuses

All melting points were measured on a hot stage apparatus (Electro thermal, Essex, UK) and are uncorrected. ^1^H NMR (300 MHz) and ^13^C NMR (75 MHz) spectra were recorded on a Bruker 300 Fourier transform spectrometer, tetramethylsilane was used as an internal standard. Chemical shifts (δ) and coupling constants (*J*) are reported in ppm and Hz, respectively. Mass spectra were recorded on an Agilent spectrometer (7000-3Q mass spectrometer at an electron impact mode with an ionization voltage of 70 eV). Infrared spectra were obtained using Bruker Tensor 27 spectrometer as KBR disks. Melting points were determined with a MEL-TEMP model 1202.

### Chemistry

#### General procedure for the synthesis of compounds 7a-e and 11a-e

The synthesis route of compounds is displayed in Fig. 2. The condensation of 2-aminopyridine **1** with appropriate ethyl 3-bromo-2-oxo propanoate **2** resulted in the formation of ethyl imidazo[1,2-a]pyridine-2-carboxylate **3**. The carbohydrazide derivative **5** was produced in the presence of an excess amount of hydrazine hydrate. Compound **7a-e** was prepared via the reaction of different aldehydes **6a-e** and **5**. Moreover, compound **5** was treated with propargyl bromide to provide prop-2-yn-1-yl imidazo[1,2-a]pyridine-2-carbohydrazide **9** which was further coupled with various benzyl azide intermediates **10a-e** through click reactions to generate **11a‑e** derivatives.

#### Synthesis of *ethyl imidazo[1,2-a]pyridine-2-carboxylate *(3)

2-Aminopyridine (**1,** 5 mmol) was first dissolved in ethanol (10 mL) and ethyl 3-bromo-2-oxo propanoate (**2**, 7.5 mmol) was added gently to the mixture. The reaction mixture was stirred at 0 °C for 1 h. The reaction was completed after 24 h; the progression of the reaction was regularly monitored by thin-layer chromatography (TLC). After completion, the solvent was evaporated. The precipitate was filtered off and washed with diethyl ether, dried, and recrystallized from ethanol to afford a creamy white solid.

Yield: 90%, Rf = 0.70 (CHCl_3_:MeOH 90:10); mp: 135–137 ^◦^C, IR (KBr, cm^−1^) υ max: 3086 (O–H), 1729 (Ester C = O); ^1^HNMR (300 MHz, Acetone-d6): δ (ppm) 8.41 (d, *J* = 6.0 Hz, 1H, imidazopyridine-H5), 8.30 (s, 1H, imidazopyridine-H), 7.44 (d, *J* = 9.0 Hz, 1H, imidazopyridine-H), 7.20 (t, J = 6.0 Hz, 1H, imidazopyridine-H), 6.84 (t, *J* = 6.0 Hz, 1H, imidazopyridine-H), 4.22 (q, 2H, COOCH_2_CH_3_), 1.23 (t, *J* = 9.0, 3H, COOCH_2_CH_3_), MS (EI) m/z: 190 (M^+^), 145, 118, 91, 78.

#### Synthesis of *imidazo[1,2-a]pyridine-2-carbohydrazide *(5)

Ethylimidazo[1,2-a]pyridine-2-carboxylate (**3,** 5 mmol) was reacted with excess amounts (3–4 mL) of hydrazine hydrate **4** for 3 h under the reflux condition. After completion, the mixture was poured into ice water (50 mL), filtered, and washed with cold water.

light creamy solid, Yield: 25%, Rf = 0.36 (CHCl_3_:MeOH; 90:10), mp: 192–194 ^◦^C. IR (KBr, cm ^−1^) υ max: 3353 and 3187 (NH2), 1660 (Amide C = O), ^1^HNMR (300 MHz,DMSO): δ_H_ (ppm) 9.53 (s, 1H, CONH), 8.58 (d, *J* = 6.0 Hz, 1H, imidazopyridine-H), 8.36 (s, 1H, imidazopyridine-H), 7.59 (d, *J* = 9.0 Hz, 1H, imidazopyridine-H), 7.33 (t, *J* = 9.0 Hz, 1H, imidazopyridine-H), 6.97 (t, *J* = *6.0* Hz, 1H, imidazopyridine-H-6), 4.48 (s, 2H, CONHNH_2_), MS (EI) m/z: 176 (M +), 145, 117, 97, 78.

### General procedure for synthesis of N´-benzylidene imidazo[1,2-a]pyridine-2-carbohydrazide derivatives (7a-e)

Title compounds **7a-e** were synthesized by adding appropriate aromatic aldehydes (1 mmol) into the solution of imidazo[1,2-a]pyridine-2-carbohydrazide ( **5**, 1 mmol) in ethanol and refluxed for 24 h. After completion of the reaction as indicated by TLC (chloroform:methanol 95:5), the reaction mixture was allowed to cool at room temperature. The precipitated product was filtered and re-crystallized from methanol.

#### Synthesis *of N´-benzylidene imidazo[1,2-a]pyridine-2-carbohydrazide *(7a)

Yield: 53%, Rf = 0.40 (CHCl_3_:MeOH 95:5), mp: 257–259 °C, IR (KBr, cm ^−1^) υ max: 3244 (Amide N–H), 1666 (Amide C = O). ^1^HNMR (300 MHz, DMSO-d_6_): δ_H_ (ppm) 11.95 (s, 1H, CONH), 8.62 (m, 2H, N = CH and imidazopyridine-H), 8.55 (s, 1H, imidazopyridine-H), 7.71 (m, 2H, Ar–H), 7.65 (d, *J* = *9.0* Hz, 1H, imidazopyridine-H), 7.42 (m, 4H, imidazopyridine-H and Ar–H), 7.03 (t, *J* = 6.0 Hz, 1H, imidazopyridine-H); ^13^CNMR (75 MHz, DMSO): δ_C_ (ppm) 158.97 (CONH), 148.49, 144.34, 138.74, 134.92, 130.49, 129.32 (2C), 128.24, 127.54 (2C), 127.34, 117.64, 116.37, 113.97.MS (EI) m/z: 263 (M +), 161, 143, 118, 97, 78.

#### Synthesis of *N´-(4-fluorobenzylidene)imidazo[1,2-a]pyridine-2-carbohydrazide *(7b)

Yield: 39%, Rf = 0.43 (CHCl_3_:MeOH 95:5), mp: 234–236 °C, IR (KBr, cm ^−1^) υ max: 3302 (Amide N–H), 1669 (Amide C = O), ^1^HNMR (300 MHz, DMSO-d_6_): δ_H_ (ppm) 11.95 (s, 1H, CONH), 8.62 (m, 2H, N = CH and imidazopyridine-H), 8.55 (s, 1H, imidazopyridine-H), 7.76 (m, 2H, Ar–H), 7.65 (d, *J* = *9.0* Hz, 1H, imidazopyridine-H), 7.39 (t, *J* = 9.0 Hz, 1H, imidazopyridine-H), 7.30 (m, 2H, Ar–H), 7.03 (t, *J* = 6.0 Hz, 1H, imidazopyridine-H); ^13^CNMR (75 MHz, DMSO): δ_C_ (ppm) 161.86 (CONH),, 159.04, 147.26, 144.41, 138.95, 131.57, 129.71, 129.60, 128.20, 127.14, 117.73 116.53, 116.38, 116.24, 113.86.MS (EI) m/z: 282 (M +), 161, 144, 118, 91, 78.

#### Synthesis of *N´-(4-chlorobenzylidene)imidazo[1,2-a]pyridine-2-carbohydrazide *(7c)

Yield: 44%, Rf = 0.44 (CHCl_3_:MeOH 95:5), mp: 226–228 °C, IR (KBr, cm ^−1^) υ max: 3441 (Amide N–H), 1656 (Amide C = O), ^1^HNMR (300 MHz, DMSO-d_6_): δH (ppm) 12.02 (s, 1H, CONH), 8.62 (m, 2H, N = CH, imidazopyridine-H-5), 8.55 (s, 1H, imidazopyridine-H-3), 7.73 (d, *J* = 9.0 Hz, 2H, Ar–H), 7.65 (d, *J* = *9.0* Hz, 1H, imidazopyridine-H), 7.53 (d, *J* = 9.0 Hz, 2H, Ar–H), 7.39 (t, *J* = 9.0 Hz, 1H, imidazopyridine-H), 7.03 (t, *J* = 6 Hz, 1H, imidazopyridine-H), ^13^CNMR (75 MHz, CDCl_3_): δ_C_ (ppm) 157.30 (CONH), 145.69, 143.29, 135.34, 131.16, 127.94 (3C), 125.85, 125.59, 116.94, 114.48, 112.95. MS (EI) m/z: 300 (M + 2, 9), 298 (M +), 161, 144, 118, 91, 78.

#### Synthesis of *N´-(4-bromobenzylidene)imidazo[1,2-a]pyridine-2-carbohydrazide *(7d)

Yield: 48%, Rf = 0.59 (CHCl3:MeOH 95:5), mp: 230–232 °C, IR (KBr, cm ^−1^) υ max: 3441 (Amide N–H), 1654 (Amide C = O),^1^HNMR (300 MHz, DMSO-d_6_): δ_H_ (ppm) 12.03 (s, 1H, CONH), 8.62 (d, *J* = 6.0 Hz, 1H, imidazopyridine-H), 8.59 (s, 1H, N = CH) 8.56 (s, 1H, imidazopyridine-H), 7.65 (m, 5H, imidazopyridine-H and Ar–H), 7.40 (m, 1H, imidazopyridine-H-7), 7.03 (t, *J* = 6.0 Hz, 1H, imidazopyridine-H); ^13^CNMR (75 MHz, DMSO): δ_C_ (ppm) 159.11 (CONH), 147.18, 144.42, 138.80, 134.24, 132.47, 132.31, 130.67, 129.37, 128.22, 127.21, 123.64, 117.73, 116.46, 113.90. MS (EI) m/z: 344 (M + 2), 342 (M^+^), 161, 144, 118, 91, 78.

#### Synthesis of *N´-(4-methylbenzylidene)imidazo[1,2-a]pyridine-2-carbohydrazide *(7e)

Yield: 38%, Rf = 0.48 (CHCl3:MeOH 95:5), mp: 180–182 °C, IR (KBr, cm ^−1^) υ max: 3432 (Amide N–H), 1653 (Amide C = O)^, 1^HNMR (300 MHz, CDCl3): δ_H_ (ppm) 10.37 (s, 1H, CONH), 8.23 (s, 1H, imidazopyridine-H), 8.15 (s, 1H, N = CH), 8.11 (d, *J* = 9.0, 1H, imidazopyridine-H), 7.64 (d, *J* = 9.0 Hz, 2H, Ar–H), 7.52 (d, *J* = 9.0 Hz, 1H, imidazopyridine-H), 7.23 (t, overlap with CDCl3, 1H, imidazopyridine-H), 7.15 (d, *J* = 9.0 Hz, 2H, Ar–H), 6.82 (t, *J* = 6.0 Hz, 1H, imidazopyridine-H), 2.31 (s, 3H, CH_3_); ^13^CNMR (75 MHz, CDCl_3_): δ_C_ (ppm) 158.11 (CONH), 148.45, 144.15, 140.93, 138.06, 130.86, 129.41(2C), 127.86 (2C), 127.04, 126.72, 117.78, 115.55, 114.02, 21.58. MS (EI) m/z: 278 (M^+^), 161, 144, 118, 91, 78.

#### Synthesis of *N´-(Prop-2-yn-1-yl) imidazo[1,2-a]pyridine-2-carbohydrazide* (9)

A mixture of imidazo[1,2-a]pyridine-2-carbohydrazide (**5**, 2 mmol), propargyl bromide (**8**, 2 mmol), and K_2_CO_3_ (2 mmol) in DMF was stirred at room temperature for 72 h. The mixture was concentrated and extraction was performed three times with chloroform: water. The chloroform layer was separated, washed with saturated NaCl solution and dried over anhydrous Na_2_SO_4_, and filtered. then the solvent was removed under reduced pressure. The product was purified by column chromatography (ethylacetate:methanol 90:10). Yield: 22%, Rf = 0.85 (CHCl_3_:MeOH 90:10), mp: 142–144 ^◦^C. IR (KBr, cm ^−1^) υ max = 3583 (NH), 3307 (amide N–H), 1711 (Amide C = O); ^1^HNMR (300 MHz, CDCl_3_): δ_H_ (ppm) 8.77 (s, 1H, CONH), 8.09 (m, 2H, imidazopyridine-H), 7.52 (d, *J* = 9.0 Hz, 1H, imidazopyridine-H), 7.23 (t, overlap with CDCl_3_, 1H, imidazopyridine-H), 6.81 (t, *J* = 9.0 Hz, 1H, imidazopyridine-H), 4.88 (s, 1H, CONHNH), 3.70 (s, 2H, CH_2_), 2.23 (s, 1H, C CH); MS (EI) m/z: 214 (M +), 161, 144, 118, 78, 39 [13, 14].

### General procedure for synthesis of N´-((1-benzyl-1H-1,2,3-triazol-4-yl)methyl)imidazo [1,2a] pyridine-carbohydrazide derivatives (11a-e)

The mixture of benzyl bromide/chloride reagent, NaN_3_, triethylamine (2 drops), water (2 mL), and tertiary butanol (2 mL) was stirred and heated at 70 °C for 1 h. After completion, the formation of benzyl azide intermediate was confirmed by TLC (petroleum ether 100%), Then the relevant benzyl azide solution was added to a solution of N´-(Prop-2-yn-1-yl)imidazo[1,2-a]pyridine-2-carbohydrazide (**9**) in water (2 mL) and tertiary butanol (2 mL). The reaction mixture was stirred in the presence of a catalytic amount of sodium ascorbate and CuSO_4_.5H_2_O (0.05 mmol) for a period of 4–7 days. After completion of the reaction, the mixture was extracted using water and chloroform mixture three times. The combined organic layers were dried over saturated NaCl solution and Na_2_SO_4_. The product was purified by plate chromatography (ethylacetate:methanol, 90:10). At last, the products were recrystallized using ethanol to get a pure compound [31].

#### Synthesis of *N´-((1-benzyl-1H-1,2,3-triazol-4-yl)methyl)imidazo[1,2-a]pyridine-2-carbohydrazide *(11a)

For this reaction, benzyl bromide (**10a**, 1.1 mmol), NaN_3_ (1 mmol), and compound **9 (**0.8 mmol) were employed as starting materials. Yield: 21%, Rf = 0.24 (EtOAc:EtOH 90:10), mp: 150–152 ^◦^C, IR (KBr, cm ^−1^) υ max: 3584 (amine N–H), 3405 (amide N–H), 1711 (amide C = O). ^1^HNMR (300 MHz, CDCl_3_): δ_H_ (ppm) 8.31(s, 1H, CONH), 8.20 (s, 1H, imidazopyridine-H), 8.10 (d, *J* = 9.0 Hz, 1H, imidazopyridine-H), 8.03 (s, 1H, triazole-H), 7.64 (m, 1H, imidazopyridine-H), 7.53` (d, *J* = 9.0 Hz, 1H, imidazopyridine-H), 7.33 (m, 3H, Ar–H), 7.24 (m, 2H, Ar–H), 6.83 (t, *J* = 9.0 Hz, 2H, imidazopyridine-H), 5.49 (s, 2H, CH_2_), 4.14 (s, 2H, CH_2_), ^13^CNMR (75 MHz, CDCl_3_): δ_C_ (ppm) 161.06 (CONH), 144.48, 143.26, 138.76,134.62, 129.00 (2C), 128.58, 128.01(2C),126.27, 125.93,123.63, 118.43, 114.51, 113.43, 54.12, 51.39. MS (EI) m/z: 345 (M +), 162, 145, 118, 91.

#### Synthesis of* N´-((1-(4-fluorobenzyl)-1H-1,2,3-triazol-4-yl)methyl)imidazo[1,2-a]pyridine-carbohydrazide *(11b)

For this reaction, 4-fluorobenzyl chloride (**10b**, 1.65 mmol), NaN_3_ (1.5 mmol), and compound **9 (**1.2 mmol) were employed. Yield: 17%, Rf = 0.20 (EtOAc:EtOH 90:10), mp: 205–207 ^◦^C, IR (KBr, cm ^−1^) υ max: 3584 (amine N–H), 3306 (amide N–H), 1710 (amide C = O), ^1^HNMR (300 MHz, CDCl_3_): δ_H_ (ppm) 8.47(s, 1H, CONH), 8.05 (d, *J* = 6.0 Hz, 1H, imidazopyridine-H-5), 8.00 (s, 1H, imidazopyridine-H), 7.62 (s, 1H, triazole-H), 7.49 (d, *J* = 9.0 Hz, 1H, imidazopyridine-H), 7.18 (t, overlap with CDCl_3_, 1H, imidazopyridine-H), 7.11 (m, 2H, Ar–H), 6.87 (t, *J* = 9.0 Hz, 2H, Ar–H), 6.79 (t, *J* = 9.0 Hz, 1H, imidazopyridine-H) 5.37 (s, 2H, CH_2_), 4.21 (s, 2H, CH_2_); ^13^CNMR (75 MHz, CDCl_3_): δ_C_ (ppm) 160.85 (CONH), 144.50, 144.13, 138.57, 130.61, 130.57, 129.88, 129.77,126.34, 126.12,123.02, 118.35, 116.04, 115.75, 114.69, 113.55, 53.33, 51.89. MS (EI) m/z: 365 (M +), 258, 213, 191, 162, 145, 118, 109, 90.

#### Synthesis of *N´-((1-(4-chlorobenzyl)-1H-1,2,3-triazol-4-yl)methyl)imidazo[1,2-a]pyridine-carbohydrazide *(11c)

For this reaction, 4-chlorobenzyl chloride (**10c**, 1.1 mmol) NaN_3_ (1 mmol), and compound **9** (0.8 mmol) were used. Yield: 23%, Rf = 0.22 (EtOAc:EtOH 90:10), mp: 193–195 °C. ^1^HNMR (300 MHz, CDCl_3_): δ_H_ (ppm) 8.44 (s, 1H, CONH), 8.05 (m, 2H, imidazopyridine-H), 7.97 (s, 1H, imidazopyridine-H), 7.63 (s, 1H, triazole-H), 7.49 (d, *J* = 9.0 Hz, 2H, Ar–H), 7.16 (t, overlap with CDCl3, 1H, imidazopyridine-H), 7.04 (d, *J* = 9.0 Hz, 2H, Ar–H), 6.78 (t, *J* = 9.0 Hz, 1H, imidazopyridine-H), 5.36 (s, 2H, CH_2_), 4.21 (s, 2H, CH_2_), ^13^CNMR (75 MHz, CDCl_3_): δ_C_ (ppm) 160.57 (CONH), 144.05, 143.56, 134.54, 133.21 (2C), 129.33 (2C), 129.17 (2C), 126.56, 123.55(2C), 117.98, 114.63, 113.84, 53.35, 51.78. MS (EI) m/z: 381 (M + 2), 379 (M^+^), 161, 145, 125, 91, 78.

#### Synthesis of *N´-((1-(4-bromobenzyl)-1H-1,2,3-triazol-4-yl)methyl)imidazo[1,2-a]pyridine-carbohydrazide *(11d)

For this reaction, 4-bromobenzyl bromide (**10d**,1.65 mmol), NaN_3_ (1.5 mmol), and compound **9 (**1.2 mmol) were used. Yield: 17%, Rf = 0.32 (EtOAc:EtOH 90:10), mp: 186–188 °C, ^1^HNMR (300 MHz, CDCl_3_): δ_H_ (ppm) 8.45 (s, 1H, CONH), 8.06 (d, *J* = 6.0 Hz, 1H, imidazopyridine-H), 7.99 (s, 1H, imidazopyridine-H), 7.63 (s, 1H, triazole-H), 7.49 (d, *J* = 9.0 Hz, 2H, imidazopyridine-H), 7.30 (d, *J* = 9.0 Hz, 2H, Ar–H), 6.97 (d, *J* = 9.0 Hz, 2H, Ar–H), 6.79 (t, *J* = 9.0 Hz, 1H, imidazopyridine-H), 5.35 (s, 2H, CH_2_), 4.21 (s, 2H, CH_2_); ^13^CNMR (75 MHz, CDCl_3_): δ_C_ (ppm) 160.71 (CONH), 144.03, 143.56, 137.78, 133.83, 133.77, 132.15, 132.08, 129.68, 129.62, 126.76, 123.76, 122.71, 117.86, 114.88, 113.91, 53.41, 51.86. MS (EI) m/z: 427 (M + 2), 425 (M^+^), 320, 251, 213, 169, 145, 118, 90.

#### Synthesis of *N´-((1-(4-methylbenzyl)-1H-1,2,3-triazol-4-yl)methyl)imidazo[1,2-a]pyridine-carbohydrazide *(11e)

For this reaction, 4-methylbenzyl chloride **10e** (4.125 mmol, MW: 140.6 g/mol) was added to NaN_3_ (3.75 mmol), and in the second part, the amount of compound **9** used was 3 mmol. Yield: 24%, Rf = 0.28 (EtOAc:EtOH 90:10), mp: 140–142 ^◦^C, IR (KBr, cm^−1^) υ max: 3682 (Amine N–H), 3306 (Amide N–H), 3018 (Ar C-H), 1710 (Amide C = O), ^1^HNMR (300 MHz, CDCl3): δ_H_ (ppm) 8.51(s, 1H, CONH), 8.04 (s, 3H, Ar–H), 7.54 (brm, 2H, Ar–H), 7.08 (brm, overlap with CDCl3, 2H, Ar–H), 6.78 (s, 2H, Ar–H), 5.64 (m, 1H, Ar–H), 5.37 (brd, 2H, CH_2_), 4.20 (s, 2H, CH_2_), 2.23 (s, 3H, CH_3_); ^13^CNMR (75 MHz, CDCl_3_): δ_C_ (ppm) 160.84 (CONH), 144.50, 143.84, 138.59, 134.72, 131.61, 129.62, 128.95, 128.57, 128.03, 126.38, 126.09, 123.19, 118.36, 114.77, 113.54, 53.94, 51.78, 46.61. MS (EI) m/z: 361 (M^+^), 347, 213, 162, 145, 118, 105, 91.

### Biological activity

#### Cytotoxicity assay

MCF-7 (human breast adenocarcinoma), HT-29 (Human colorectal adenocarcinoma), K562 (human chronic myelogenous leukemia), and Vero (kidney epithelial cells of African green monkey) cells were obtained from Iranian Biological Resource Center, Tehran, Iran. MCF-7, K562, and Vero cells were cultured in RPMI 1640 medium containing 10% heat-inactivated fetal bovine serum (FBS) and 1% penicillin/streptomycin, while HT-29 cells were grown in DMEM low glucose, containing 20% heat-inactivated FBS and 1% penicillin/streptomycin. All cells were grown in monolayer culture at 37 °C in a humidified incubator with 5% CO_2_.

Assessment of cell viability after exposure of cancer and non-cancer cells to synthetic compounds was performed using the MTT (3-(4,5-dimethylthiazol-2-yl)-2,5-diphenyltetrazolium bromide) reduction assay [31, 32]. Cells were plated in 96-well microplates at densities of 30,000 cells/mL (100 μL in each well). The plates were incubated overnight at 37 °C and then 50 μL of the growth medium was replaced with the same amount of fresh medium containing 3–4 different concentrations of synthesized analogs at the final concentrations of 10–100 µM. Test compounds were initially dissolved in DMSO and then diluted in the growth medium. The upper limit of DMSO concentration in each well was 0.5%. After 72 h of incubation, 80 μL of the solution in each well was substituted with the same quantity of the growth medium comprising of MTT solution at 0.5 mg/mL. Cells were subsequently incubated at 37 °C for 4 h and then 80 μL of the solution in each well was removed. The introduction of DMSO (200 μL) to each well caused the formazan crystals formed inside the viable cells to dissolve. The process lasted 1.5 h (1 h of incubation and 30 min of shaking). The optical absorbance of the final solution was quantified at the wavelength of 570 nm using a microplate reader. IC_50_ values for all derivatives were calculated using CurveExpert 1.34 software. To prove the reliability of the results, we repeated each experiment 3 to 5 times.

#### Cell cycle analysis

The analysis of cells in different phases of the cell cycle and the sub-G1 phase were monitored using RNase/propidium iodide (PI)-based assessment of the cell cycle by flow cytometry. MCF-7 cells were seeded in 12-well plates (1 × 10^5^ cells/well) and after being incubated overnight to allow cell attachment, were treated with different concentrations of **7d** (20, 50, and 100 nM) for 48 h. At the end of the incubation, the culture media in each well were collected and the cells were washed with PBS. Then, they were fixed with 70% ethanol overnight at −20 °C. After 24 h, the fixed cells were washed with PBS and subsequently stained with DNA staining solution (PI 20 μg/mL and RNase 200 μg/mL) at room temperature for 30 min in the dark. Ten thousand cells of each sample were analyzed using a FACS Calibur flow cytometer (BD Biosciences) and the percentage of the cells in sub-G1, G0/G1, S, and G2/M phases were calculated using CellQuest (BD, USA) software [33].

#### Determination of apoptosis by Hoechst staining

Hoechst 33,258 staining assay was used to determine apoptosis induction in cancer cells. Hoechst 33,258 is a fluorescent dye that binds to minor grooves in DNA and is commonly used to determine the characteristic features of apoptosis including condensation and fragmentation, etc. in the cell’s nuclei. MCF-7 cells were cultured in 6-well plates at a density of 10^5^ cells/ml and exposed to 100 µM of compound **7d** for 72 h. The growth medium was completely removed at the end of the incubation time and the cells were fixed with 1 ml of 4% cold freshly prepared paraformaldehyde (PFA) and incubated for 20 min. Then, the cells were washed twice with PBS and incubated with 1 ml Hoechst 33,258 2.5 µg/ml for 30 min at room temperature in the dark. In the end, the cells were washed twice with PBS and imaged with a fluorescence microscope (Nikon model DS-Ri2).

### Computational studies

#### Target prediction

The probable targets of the synthesized compounds with the superior cytotoxic potential against MCF-7 and HT-29 cell lines (**7d** and **9**) were predicted using the SuperPred webserver. SuperPred employs a machine learning approach that provides a comprehensively filtered dataset which is appropriate for proper target predictions [34]. After the prediction of the probable targets, they were evaluated to determine if they were involved in cancer pathways or not. The evaluation was conducted using the Cancer Gene Census (CGC) which is an expert-curated description of the genes driving human cancer [35]. The final obtained protein was further investigated using the molecular docking study and molecular dynamics simulation.

#### Molecular docking study

The chemical activities of top-ranked synthesized compounds were estimated against the determined target. The structure of platelet-derived growth factor receptor α (PDGFRA) in the resolution of 1.9 Å (PDB ID: 6JOL) with X-RAY diffraction method was retrieved from the PDB database (http://www.rcsb.org/pdb), and the protein preparation module of the Schrödinger Suite was used for the preparation of the structures. Accordingly, the missing hydrogen atoms were added and the water molecules were removed. Afterward, an H-bond network was produced and finally, the system was minimized by implementing the OPLS force field. To obtain more trustworthy results, the active binding site of the protein was determined employing the SiteMap of Schrödinger [36], and a receptor grid was generated around the active site. The synthesized compounds were generated using the NCI Online SMILES Translator (https://cactus.nci.nih.gov/translate/) and exported as SDF files. The accurate protonation states for ligands were generated by using the LigPrep module of Schrödinger [37]. Ultimately, the molecular docking was operated using the Glide of Schrödinger suites. The output structures of LigPrep and the generated receptor grid were utilized as the inputs for Glide. The standard precision (SP) and extra precision (XP) docking calculations were conducted to obtain the appropriate pose of the ligand, and the values of the scaling factor and partial charge cutoff were set to 0.80 and 0.15, respectively.

#### Molecular dynamics (MD) simulation

The interactions between the selected compounds (**7d** and **9**) and PDGFRA were further assessed dynamically. Desmond of Schrödinger was used to conduct the MD simulation. The complex system obtained from the docking calculations was used for the MD. The simulation was performed in an orthorhombic box and the solvent model of transferable intermolecular potential with 3 points (TIP3P) was chosen for the simulation [38]. The proper numbers of Na + /Cl − ions with a salt concentration of 0.15 M were used to neutralize the system employing the system setup of Schrödinger. The simulation was then accomplished for 100 ns with the default relaxation protocol of software and the constant number of atoms, pressure, and temperature (NPT) ensemble. The Nose–Hoover protocol was used to set the temperature to 310.15 K (37 °C), and the pressure was adjusted to 1 atm employing isotropic scaling [39, 40].

### Supplementary Information

**Additional file 1: Figure S1.** ^1^HNMR data of (3). **Figure S2.** ^1^HNMR data of (5). **Figure S3.** ^1^HNMR data of (9). **Figure S4.** ^1^HNMR data of (7a). **Figure S5.** ^13^CNMR data of (7a). **Figure S6.** ^1^HNMR data of (7b). **Figure S7.** ^13^CNMR data of (7b). **Figure S8.** ^1^HNMR data of (7c). **Figure S9.** ^13^CNMR data of (7c). **Figure S10.** ^1^HNMR data of (7d). **Figure S11.** ^13^CNMR data of (7d). **Figure S12.** ^1^HNMR data of (7e). **Figure S13.** ^13^CNMR data of (7e). **Figure S14.** ^1^HNMR data of (11a). **Figure S15.** ^13^CNMR data of (11a). **Figure S16.** ^1^HNMR data of (11b). **Figure S17.** ^13^CNMR data of (11b). **Figure S18.** ^1^HNMR data of (11c). **Figure S19.** ^13^CNMR data of (11c). **Figure S20.** ^1^HNMR data of (11d). **Figure S21.** ^13^CNMR data of (11d). **Figure S22.** ^1^HNMR data of (11e). **Figure S23.** ^13^CNMR data of (11e). **Figure S24.** MS data of (3). **Figure S25.** MS data of (5). **Figure S26.** MS data of (9). **Figure S27.** MS data of (7a). **Figure S28.** MS data of (7b). **Figure S29.** MS data of (7c). **Figure S30.** MS data of (7d). **Figure S31.** MS data of (7e). **Figure S32.** MS data of (11a). **Figure S33.** MS data of (11b). **Figure S34.** MS data of (11c). **Figure S35.** MS data of (11d). **Figure S36.** MS data of (11e).

## Data Availability

All data generated or analyzed during this study are included in its supplementary information files and can be made available upon reasonable request.
