# Supplementary material for: Synthesis and cytotoxic activity evaluation of novel imidazopyridine carbohydrazide derivatives
Source: BMC Chem. 2024 Jan 6;18(1):6. doi: 10.1186/s13065-023-01073-3 (PMC10770970; doi:10.1186/s13065-023-01073-3)
Supplement: Supplementary file 1 — Additional file 1: Figure S1. 1HNMR data of (3). Figure S2. 1HNMR data of (5). Figure S3. 1HNMR data of (9). Figure S4. 1HNMR data of (7a). Figure S5. 13CNMR data of (7a). Figure S6. 1HNMR data of (7b). Figure S7. 13CNMR data of (7b). Figure S8. 1HNMR data of (7c). Figure S9. 13CNMR data of (7c). Figure S10. 1HNMR data of (7d). Figure S11. 13CNMR data of (7d). Figure S12. 1HNMR data of (7e). Figure S13. 13CNMR data of (7e). Figure S14. 1HNMR data of (11a). Figure S15. 13CNMR data of (11a). Figure S16. 1HNMR data of (11b). Figure S17. 13CNMR data of (11b). Figure S18. 1HNMR data of (11c). Figure S19. 13CNMR data of (11c). Figure S20. 1HNMR data of (11d). Figure S21. 13CNMR data of (11d). Figure S22. 1HNMR data of (11e). Figure S23. 13CNMR data of (11e). Figure S24. MS data of (3). Figure S25. MS data of (5). Figure S26. MS data of (9). Figure S27. MS data of (7a). Figure S28. MS data of (7b). Figure S29. MS data of (7c). Figure S30. MS data of (7d). Figure S31. MS data of (7e). Figure S32. MS data of (11a). Figure S33. MS data of (11b). Figure S34. MS data of (11c). Figure S35. MS data of (11d). Figure S36. MS data of (11e). [file 13065_2023_1073_MOESM1_ESM.docx]

**Synthesis and Cytotoxic Activity Evaluation of Novel Imidazopyridine Carbohydrazide Derivatives**

Maryam Firouzi,^1,2^ Zahra Haghighijoo^1^, Masoomeh Eskandari^1^, Maryam Mohabbati^1^, Ramin Miri^1^, Mohammad Hasan Jamei^1,2^, Alireza Poustforoosh^1^, Somayeh Nazari^1^, Omidreza Firuzi^1^, Mehdi Khoshneviszadeh^1^,* Najmeh Edraki*

^1^Medicinal and Natural Products Chemistry Research Center, Shiraz University of Medical Sciences, Shiraz, Iran

^2^Department of Medicinal Chemistry, Faculty of Pharmacy, Shiraz University of Medical Sciences, Shiraz, Iran.

**NMR spectroscopy results:**


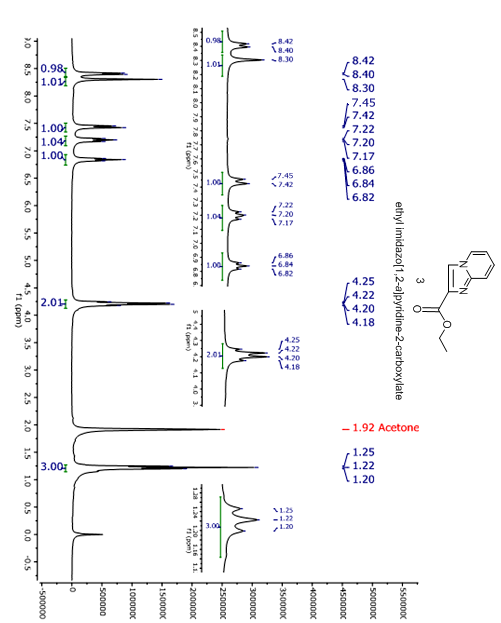


Figure S1. ^1^HNMR data of (3)


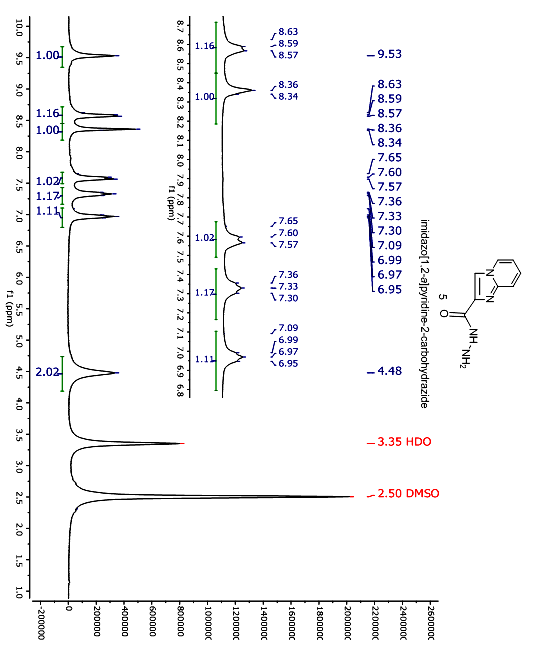


Figure S2. ^1^HNMR data of (5)


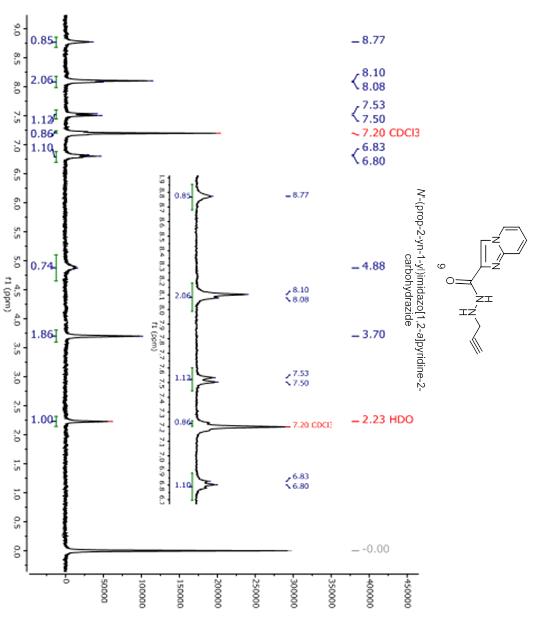


Figure S3. ^1^HNMR data of (9)


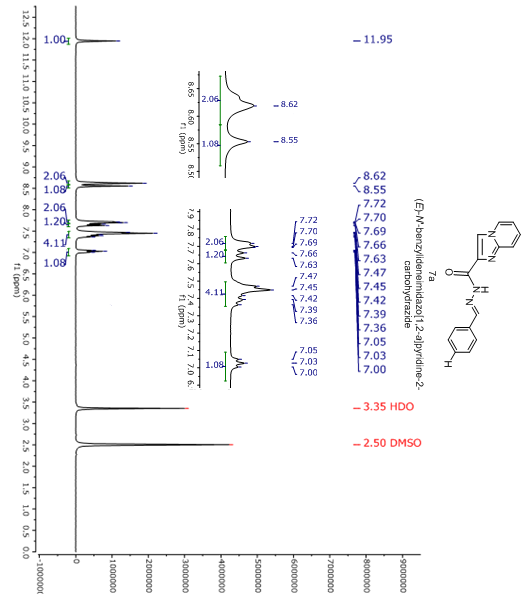


Figure S4. ^1^HNMR data of (7a)


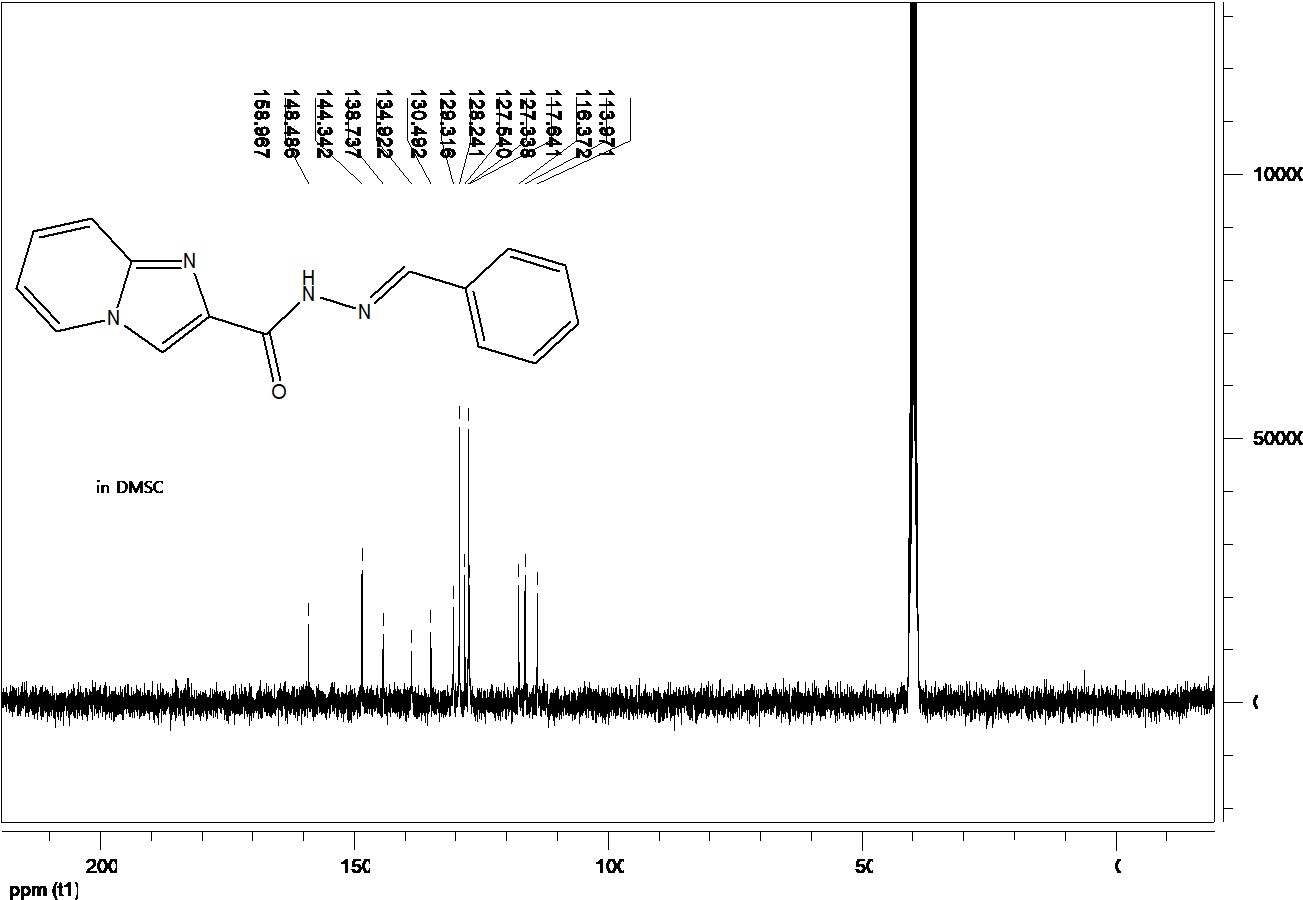


Figure S5. ^13^CNMR data of (7a)


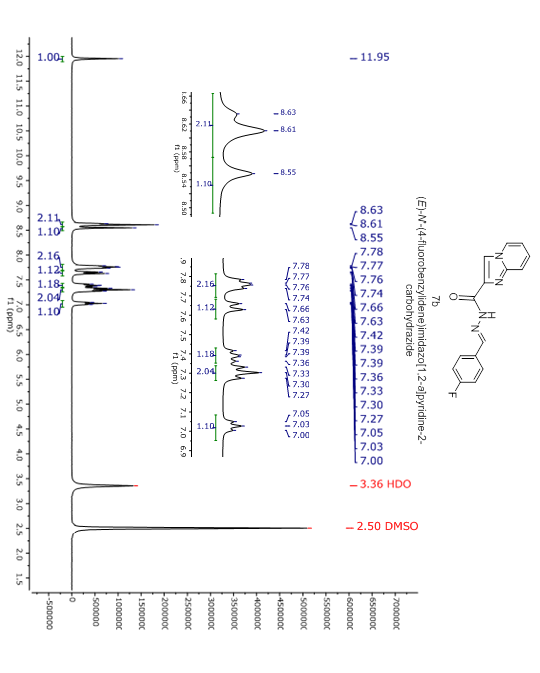


Figure S6. ^1^HNMR data of (7b)


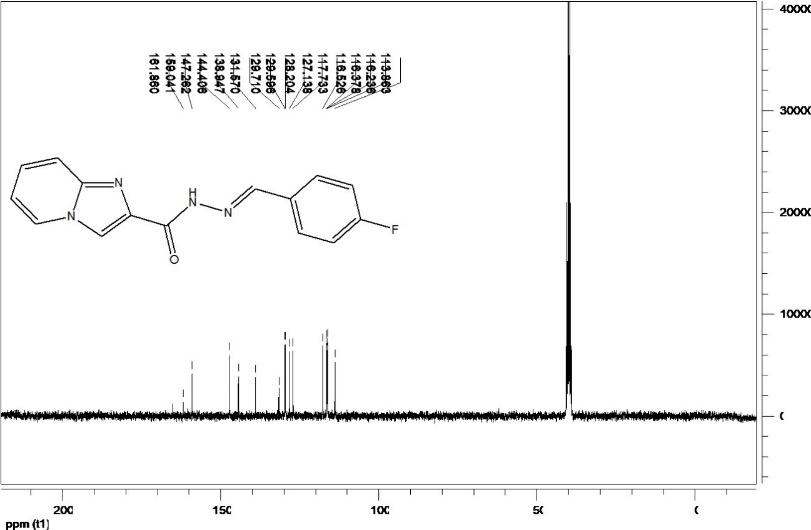


Figure S7. ^13^CNMR data of (7b)


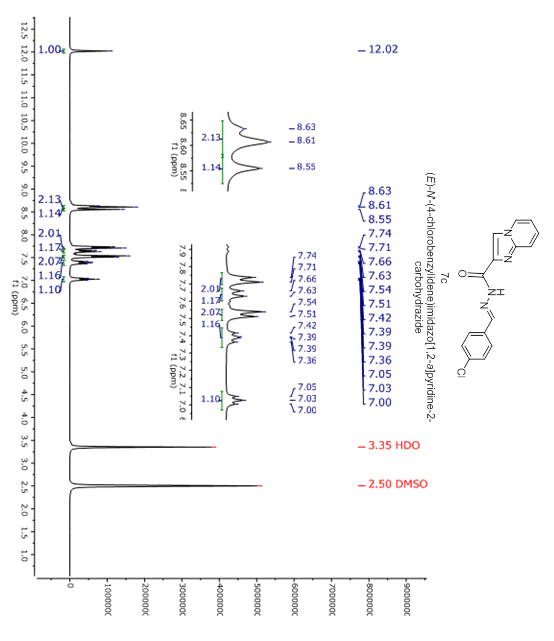


Figure S8. ^1^HNMR data of (7c)


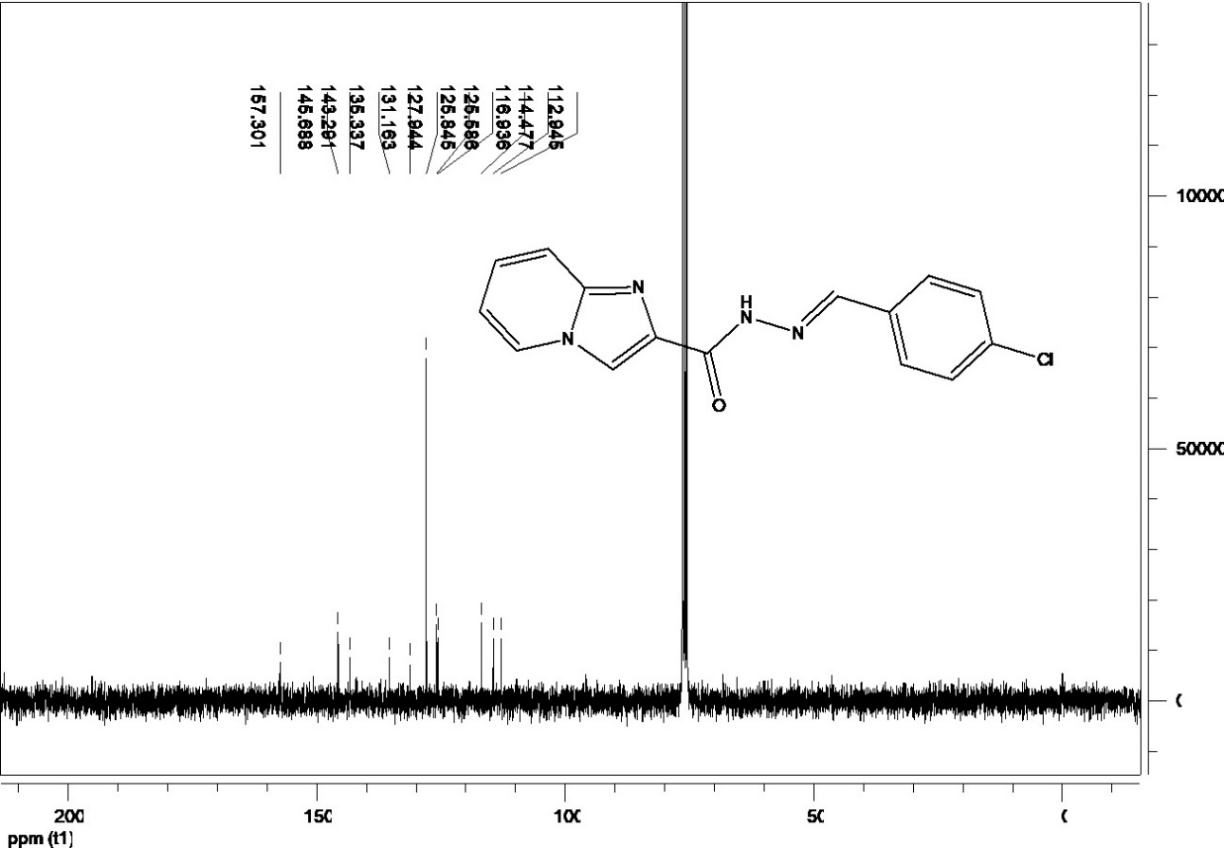


Figure S9. ^13^CNMR data of (7c)


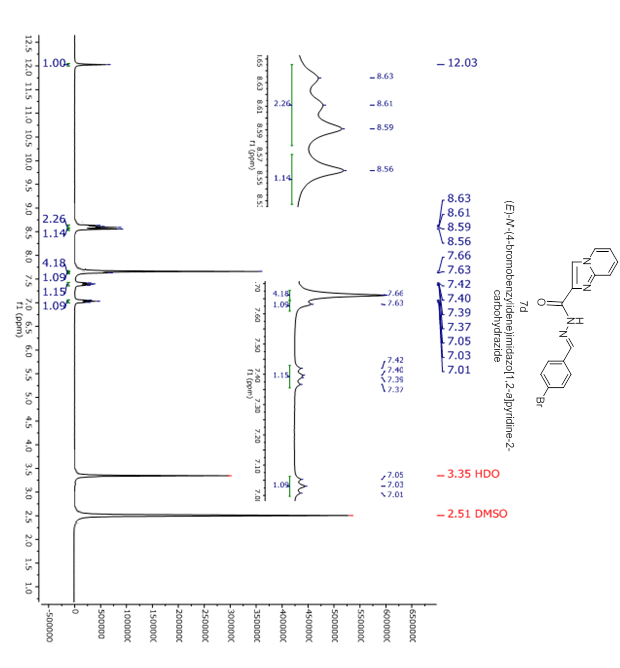


Figure S10. ^1^HNMR data of (7d)


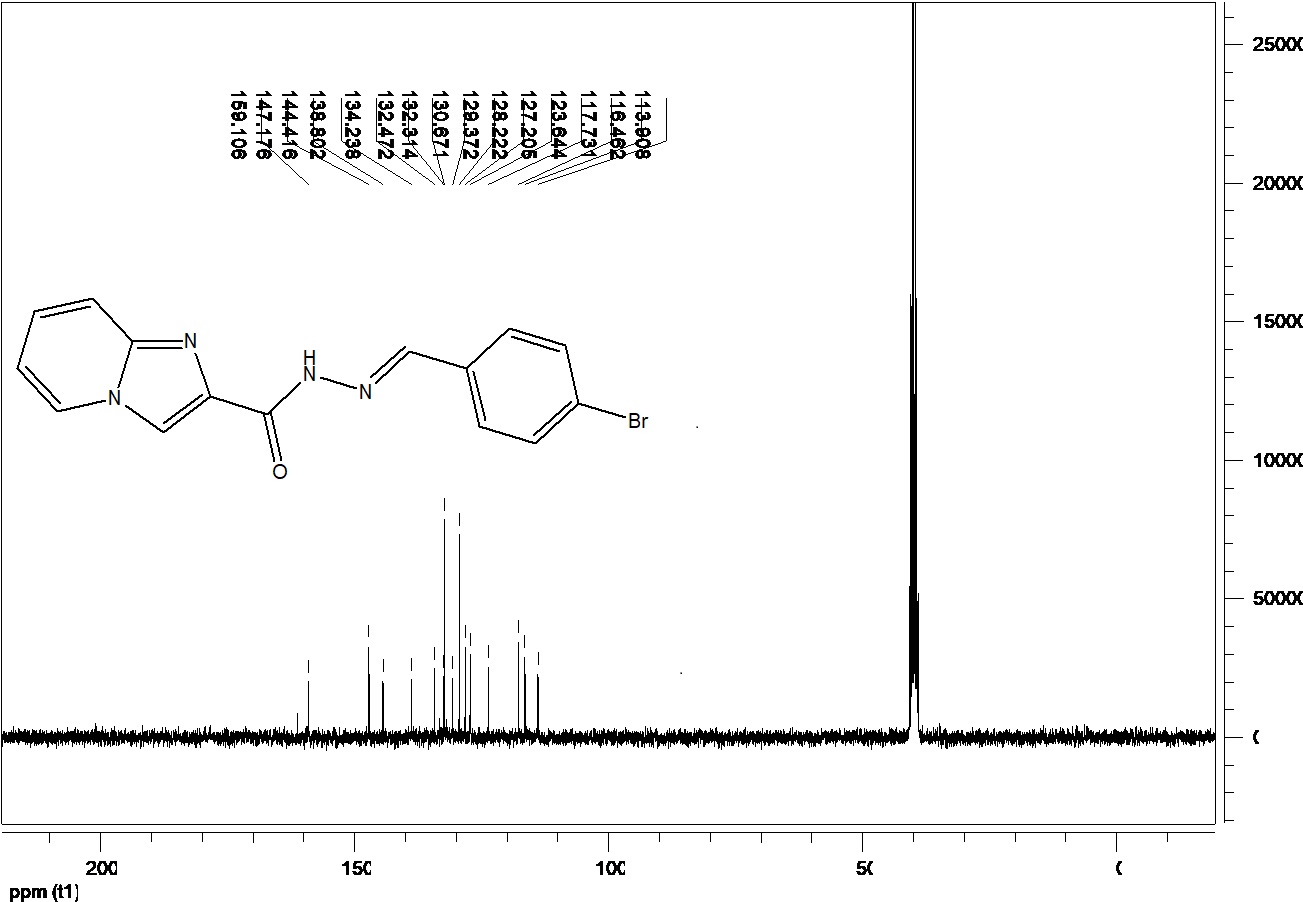


Figure S11. ^13^CNMR data of (7d)


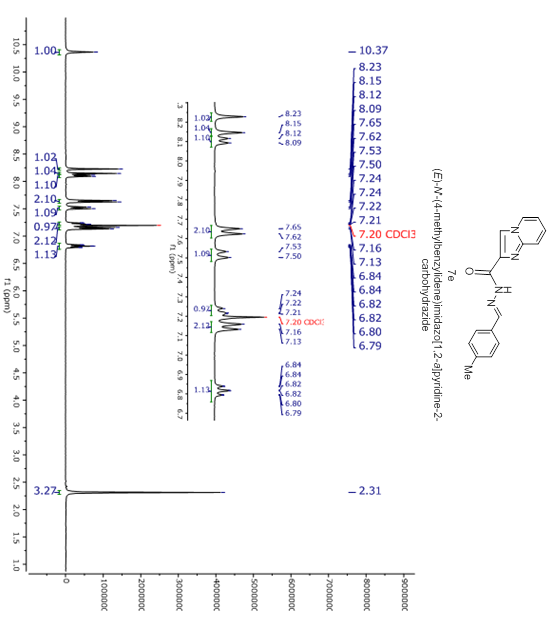


Figure S12. ^1^HNMR data of (7e)


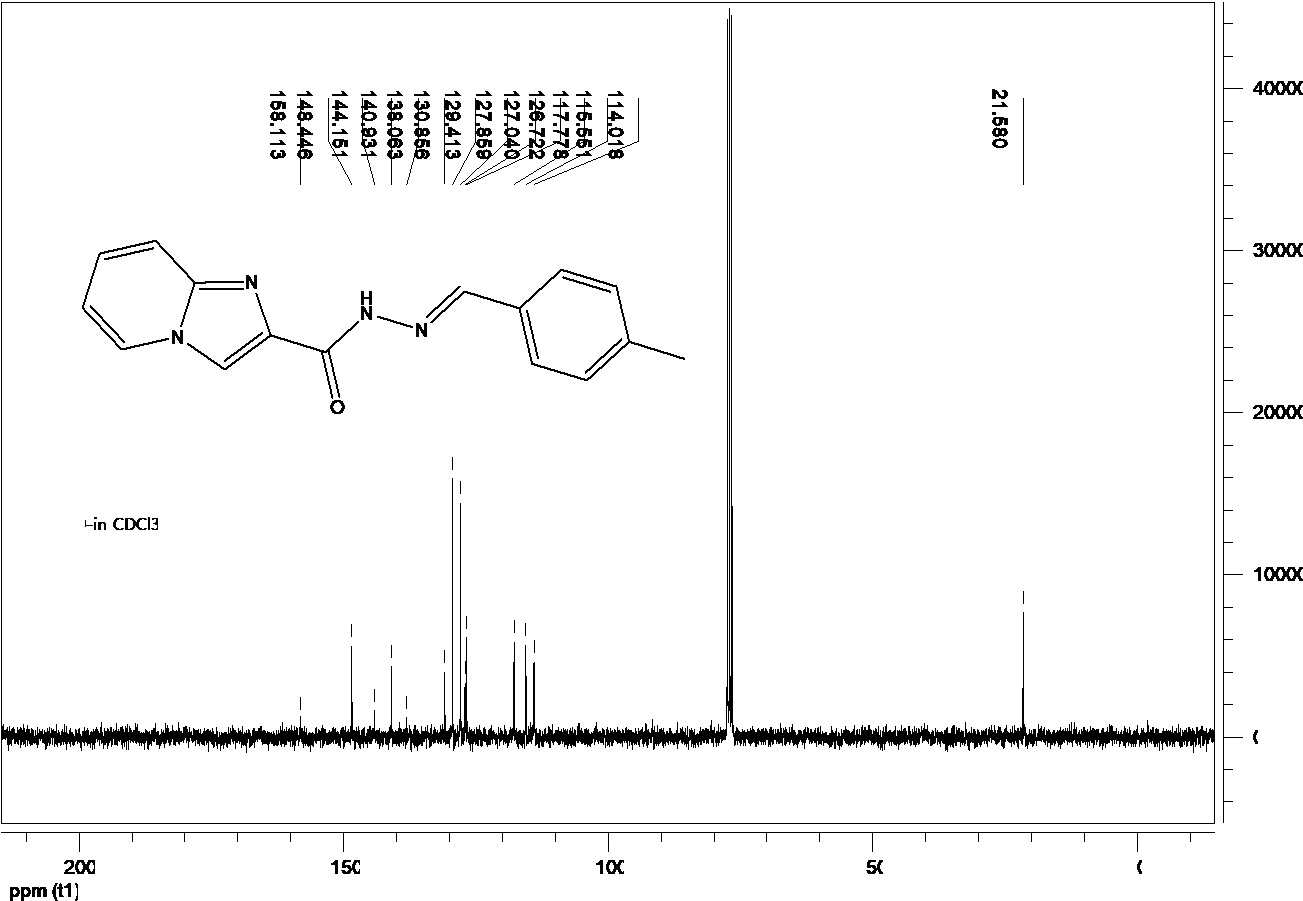


Figure S13. ^13^CNMR data of (7e)


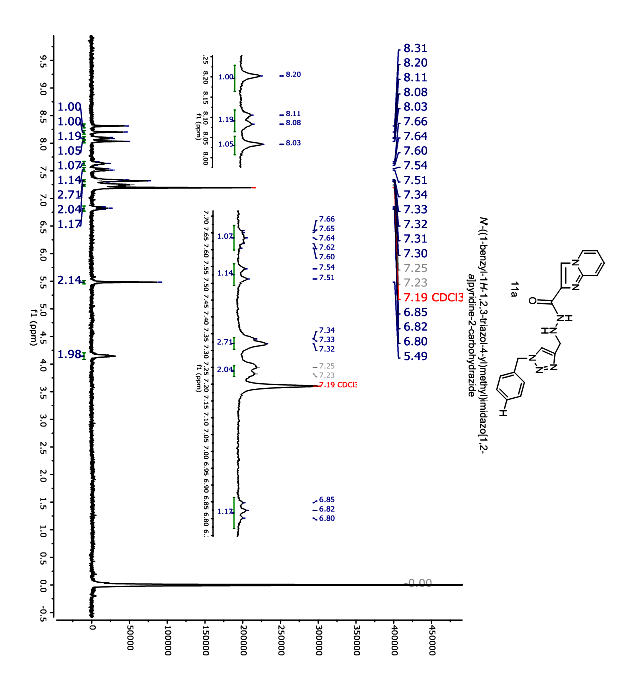


Figure S14. ^1^HNMR data of (11a)


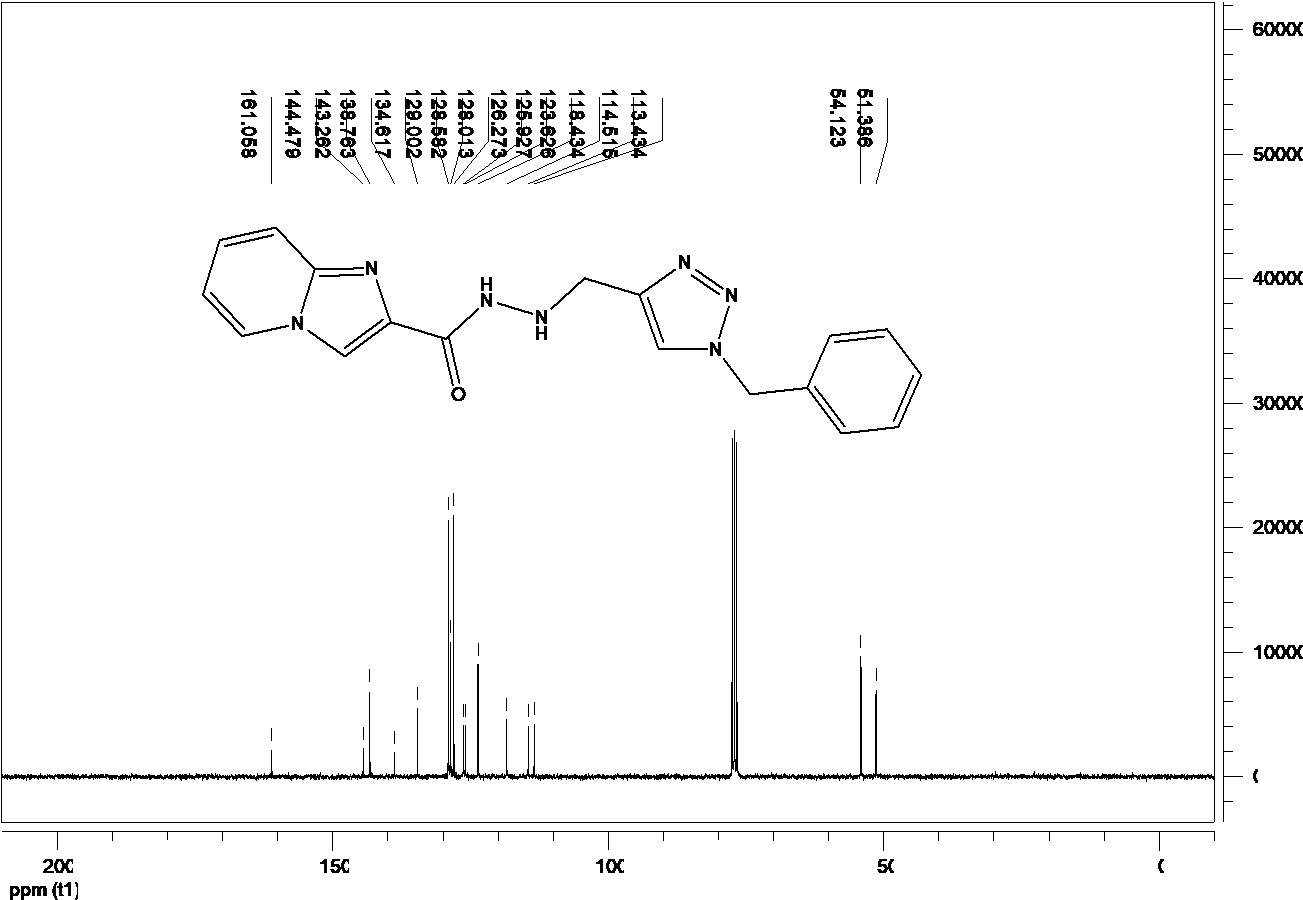


Figure S15. ^13^CNMR data of (11a)


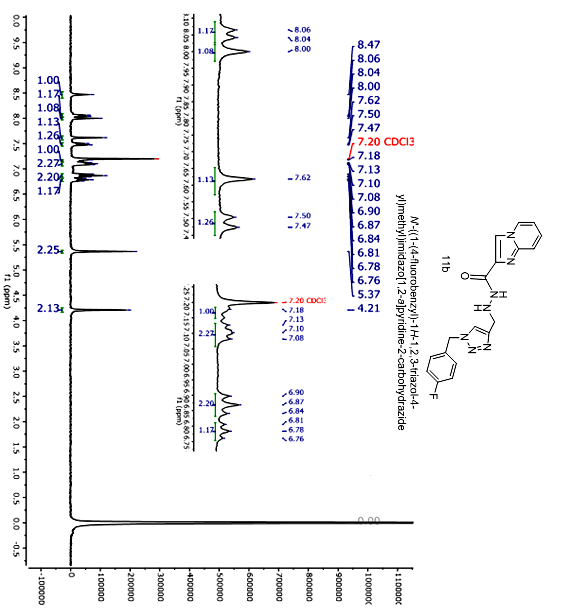


Figure S16. ^1^HNMR data of (11b)


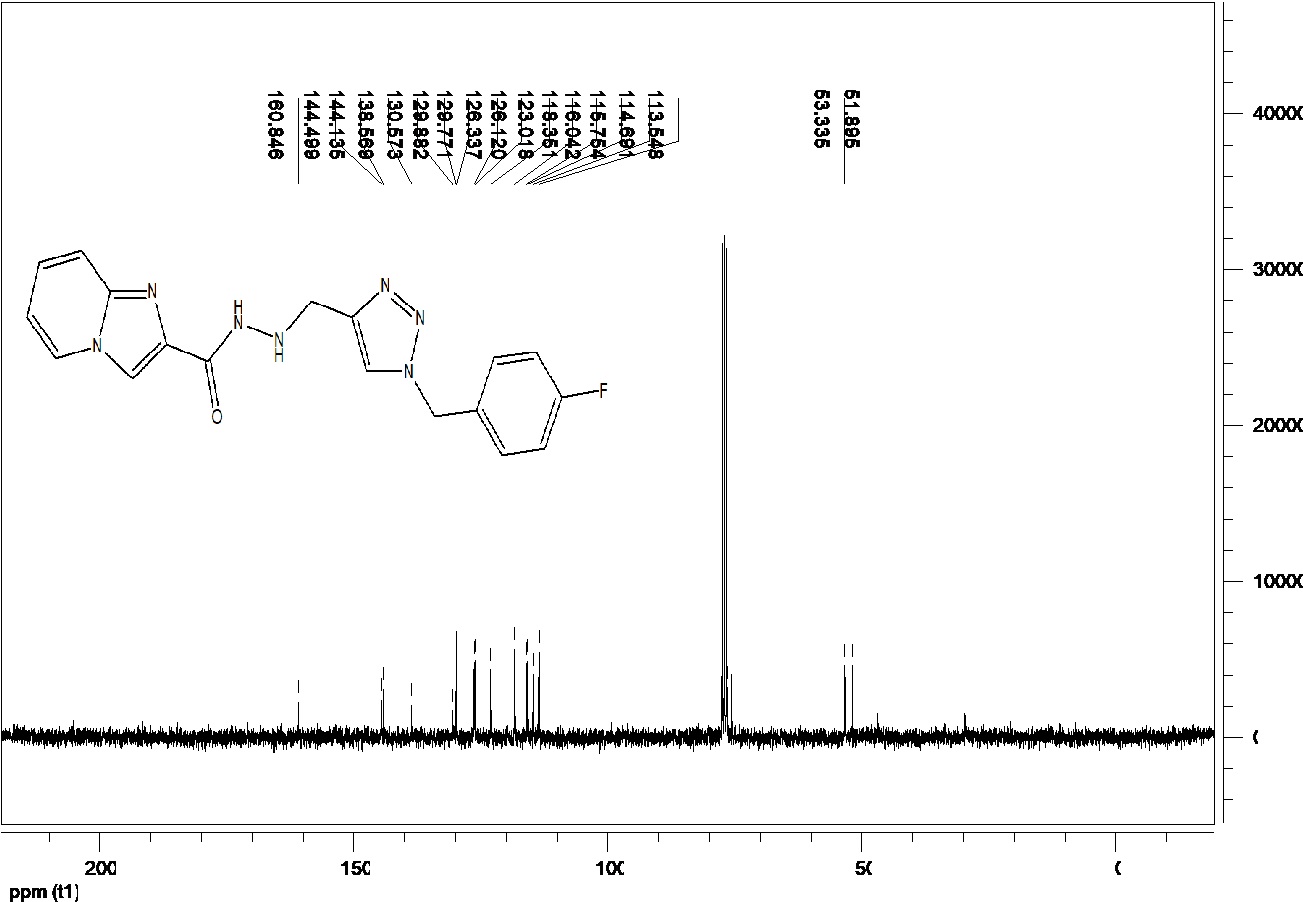


Figure S17. ^13^CNMR data of (11b)


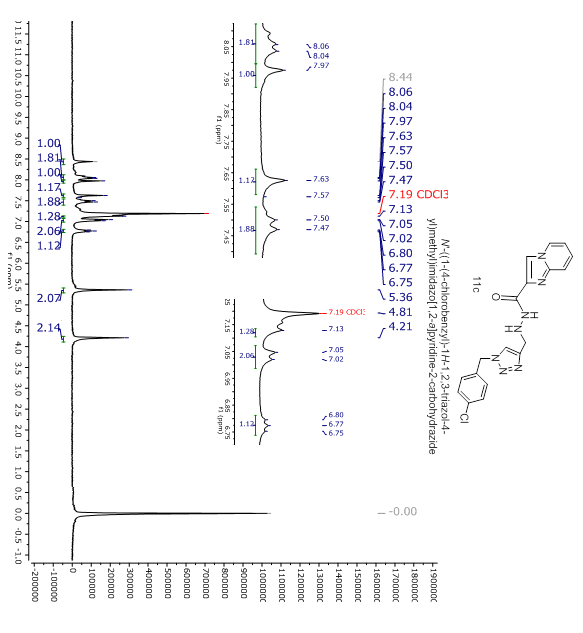


Figure S18. ^1^HNMR data of (11c)


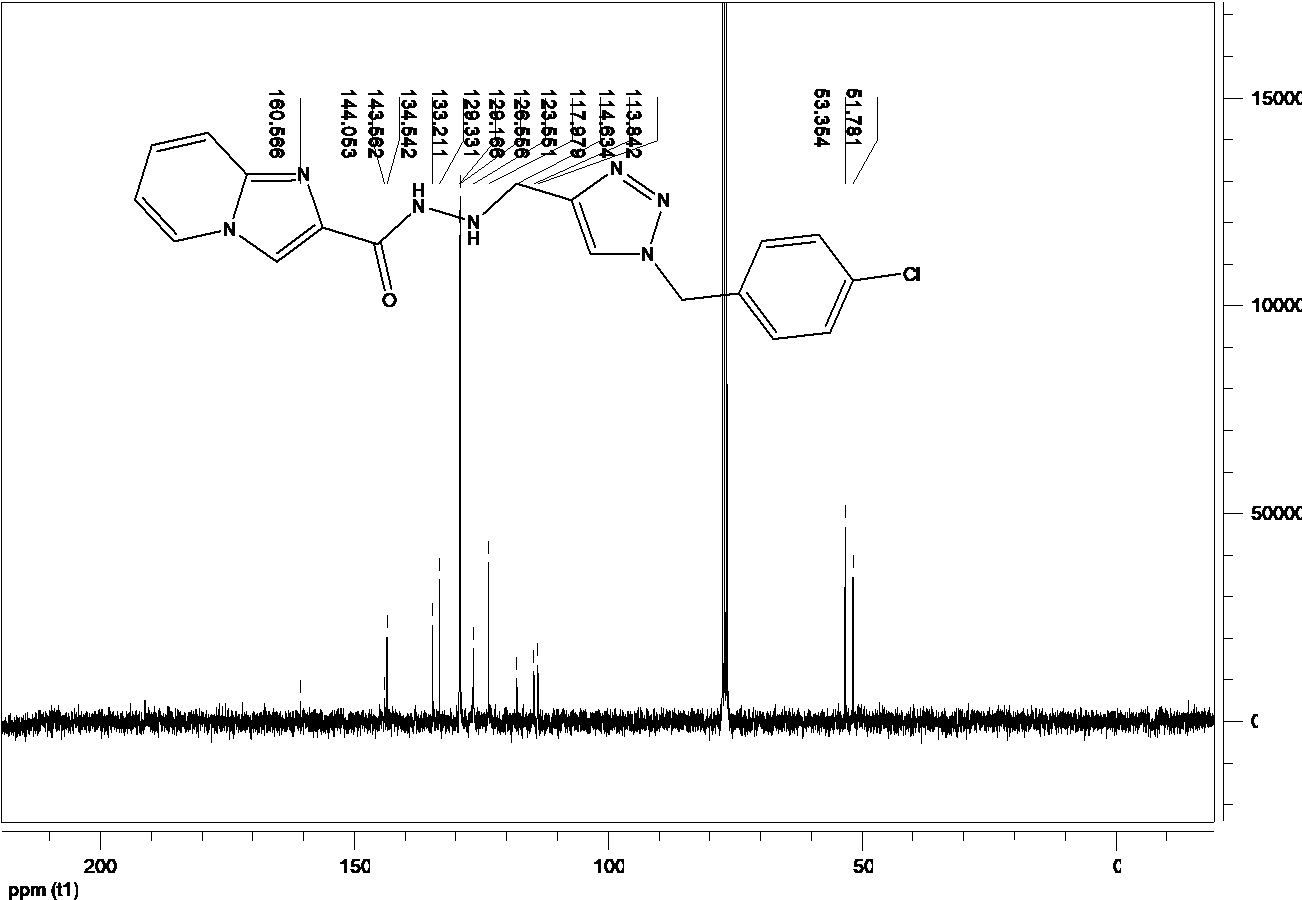


Figure S19. ^13^CNMR data of (11c)


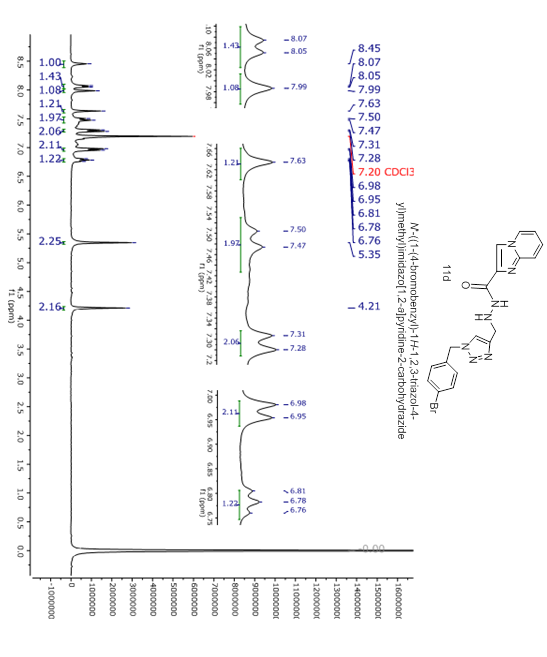


Figure S20. ^1^HNMR data of (11d)


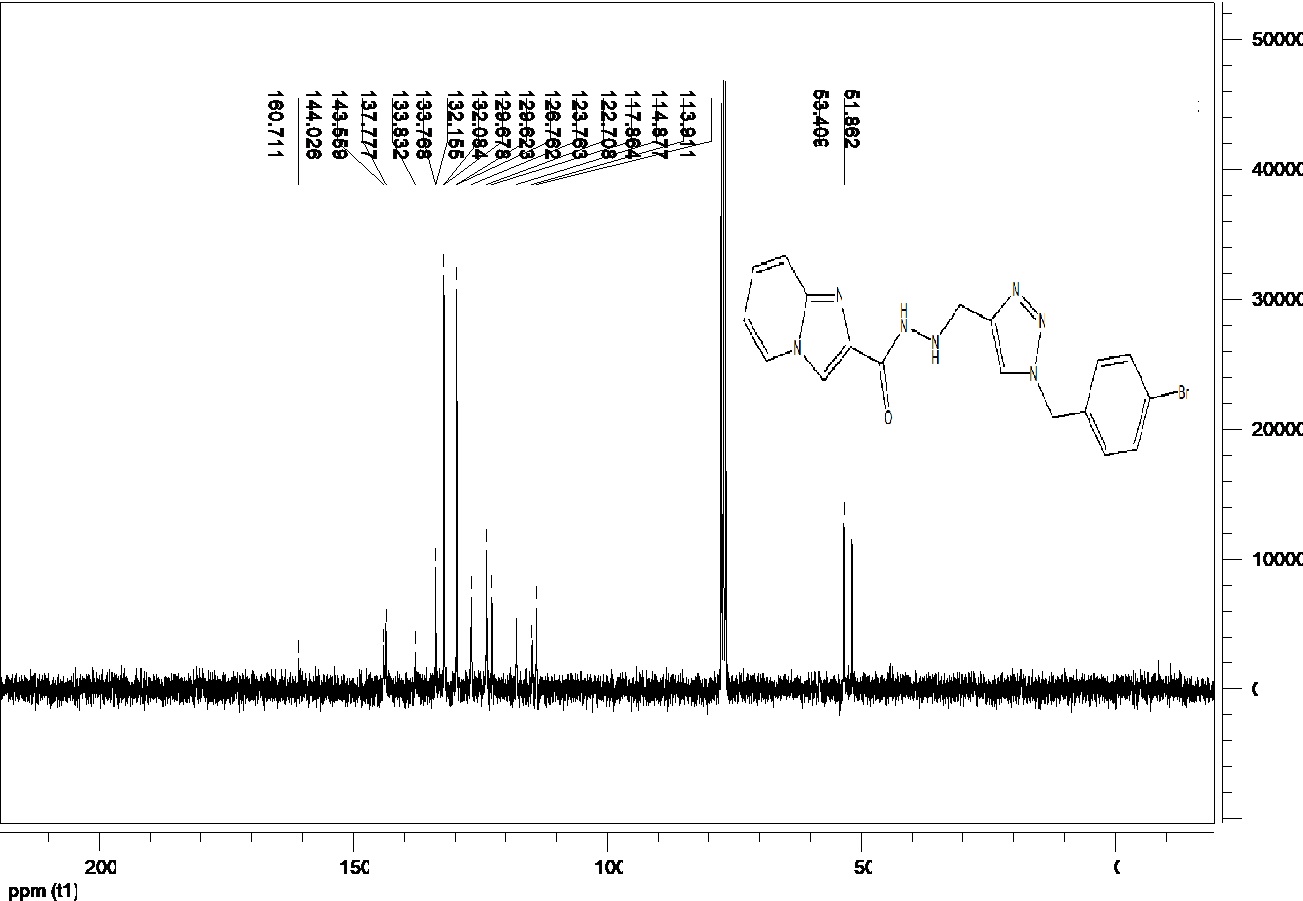


Figure S21. ^13^CNMR data of (11d)


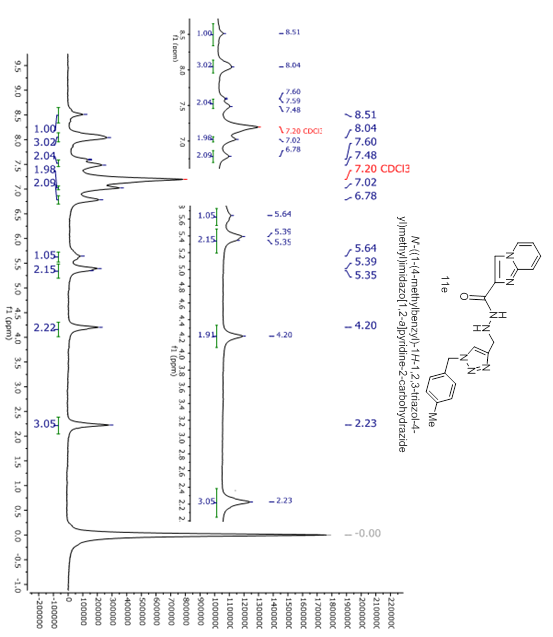


Figure S22. ^1^HNMR data of (11e)

N´-((1-(4-fluorobenzyl)-1H-1,2,3-triazol-4-yl)methyl)imidazo[1,2-a]pyridine-carbohydrazide (11b)


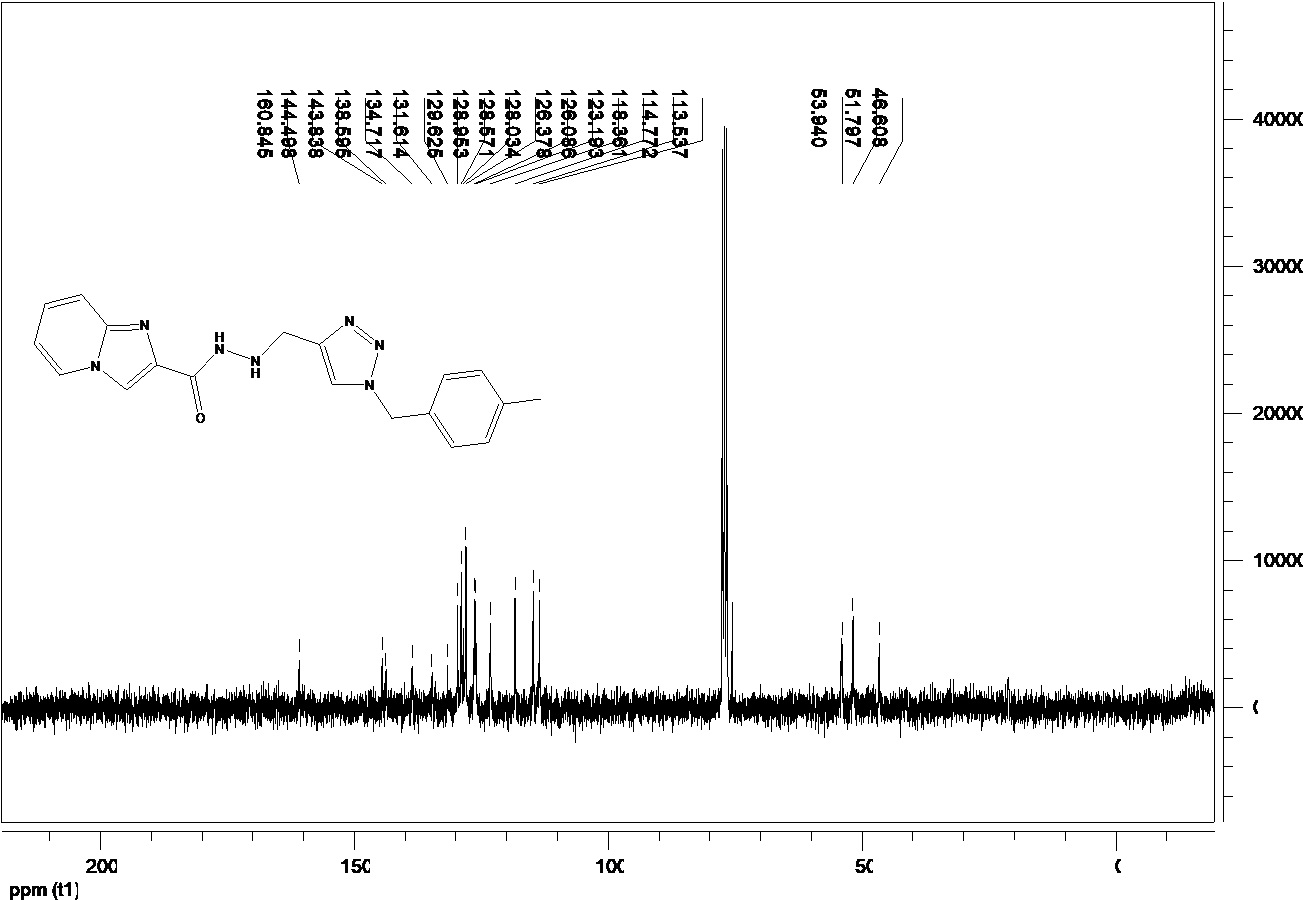


Figure S23. ^13^CNMR data of (11e)

**MS spectrometry results:**


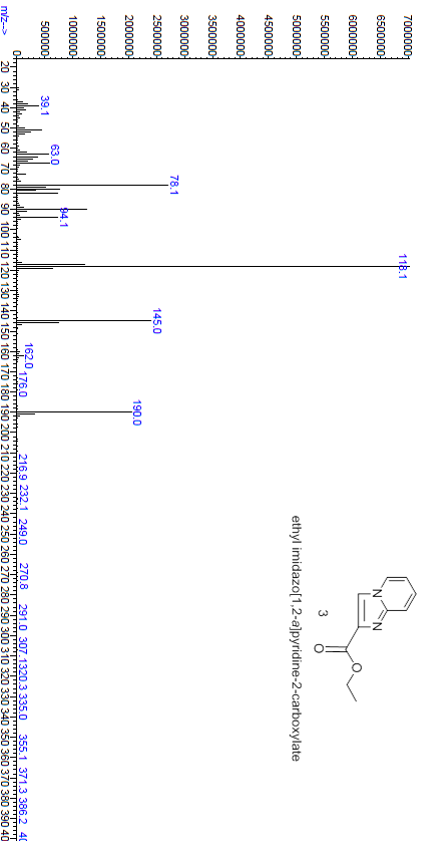


Figure S24. MS data of (3)


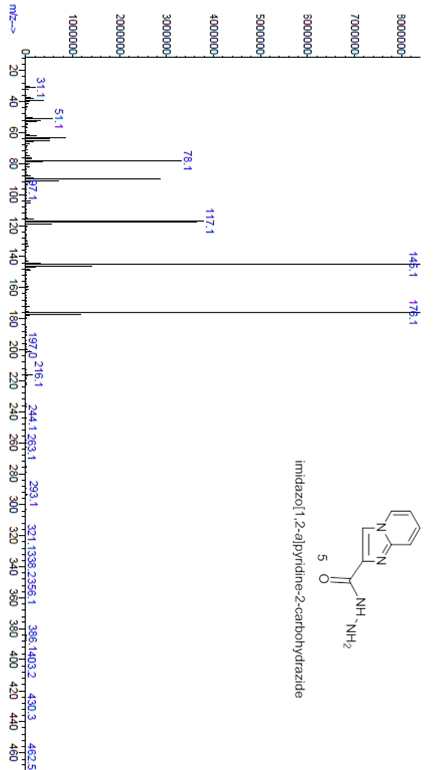


Figure S25. MS data of (5)


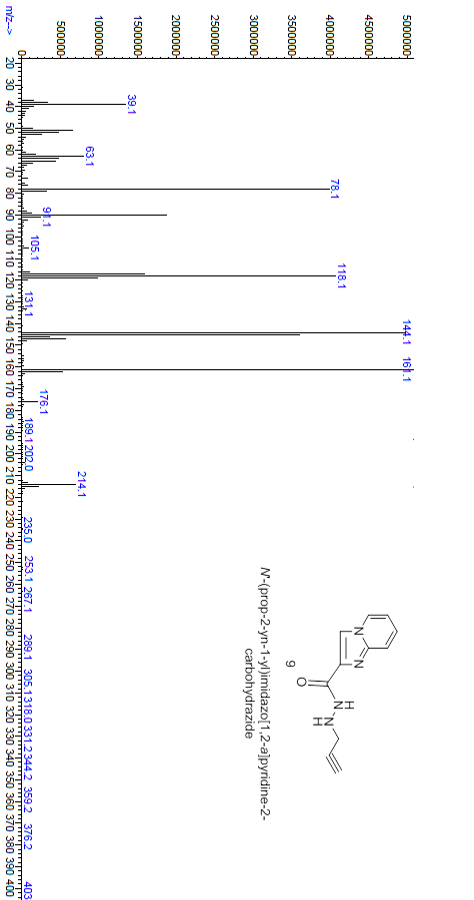


Figure S26. MS data of (9)


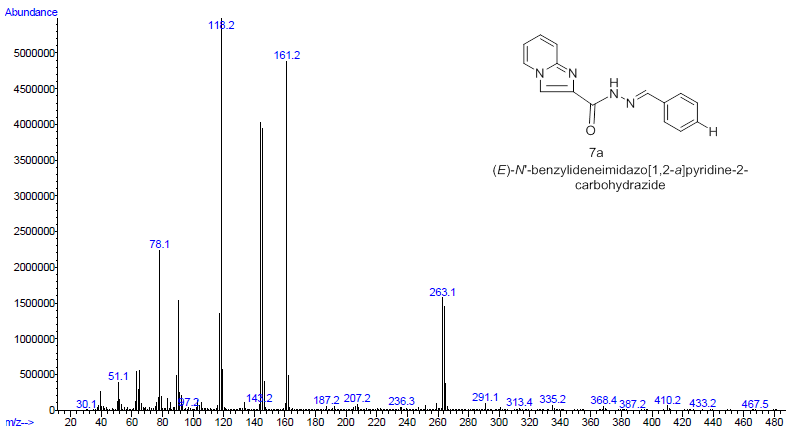


Figure S27. MS data of (7a)

**Chemical structure of major fragments of 7a**


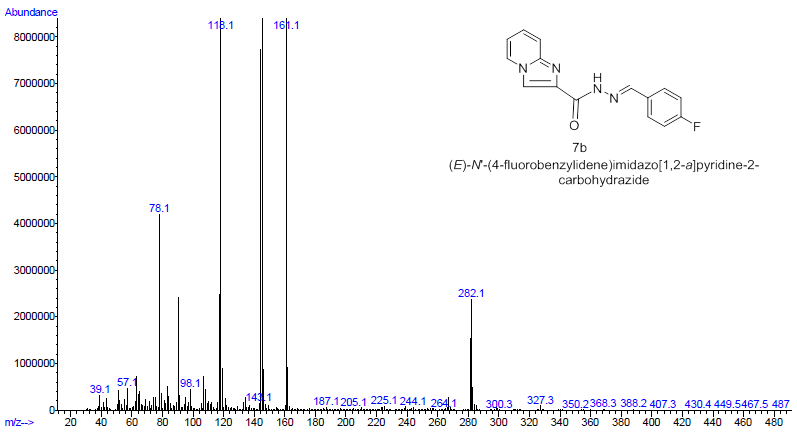


Figure S28. MS data of (7b)


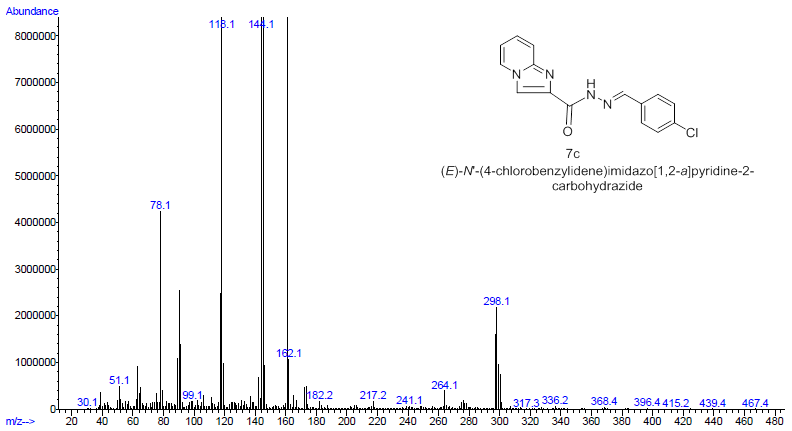


Figure S29. MS data of (7c)


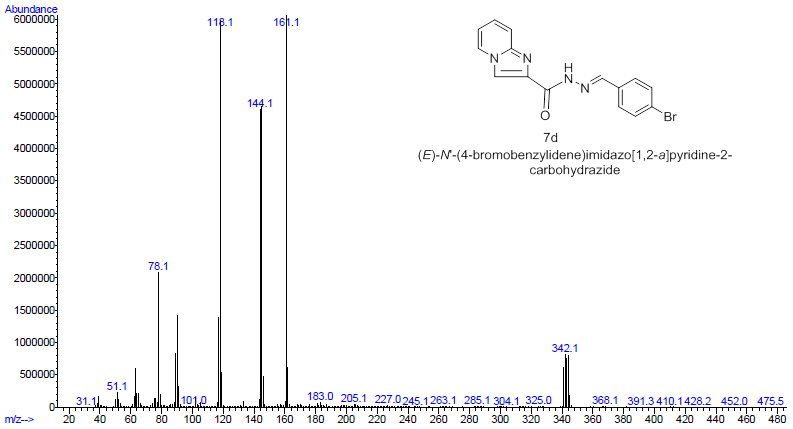


Figure S30. MS data of (7d)


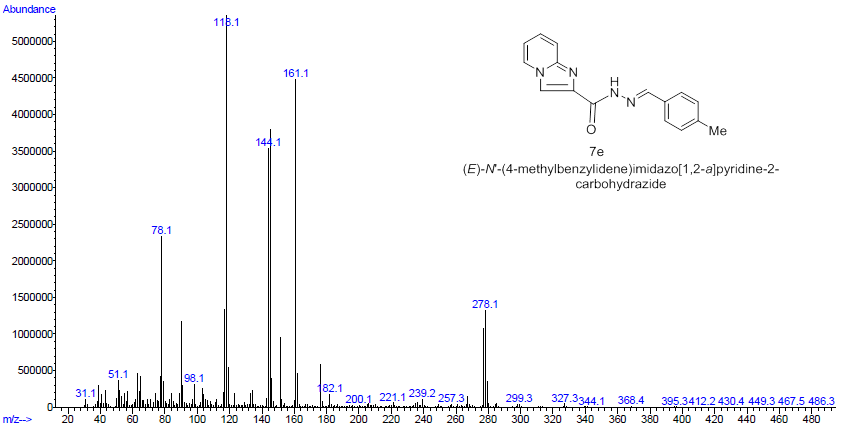


Figure S31. MS data of (7e)


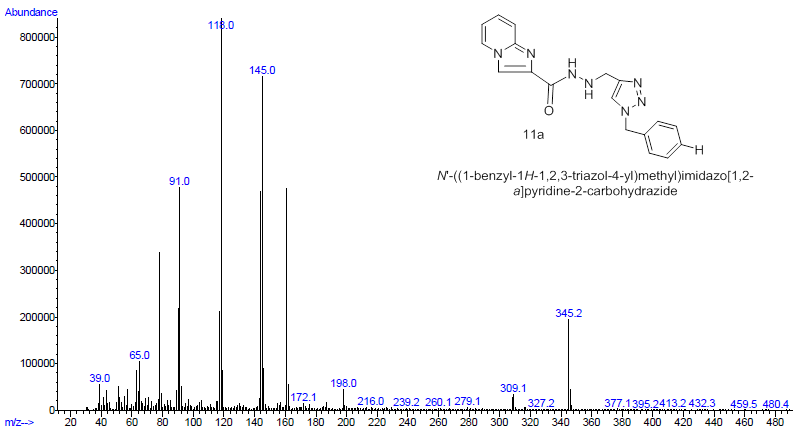


Figure S32. MS data of (11a)

**
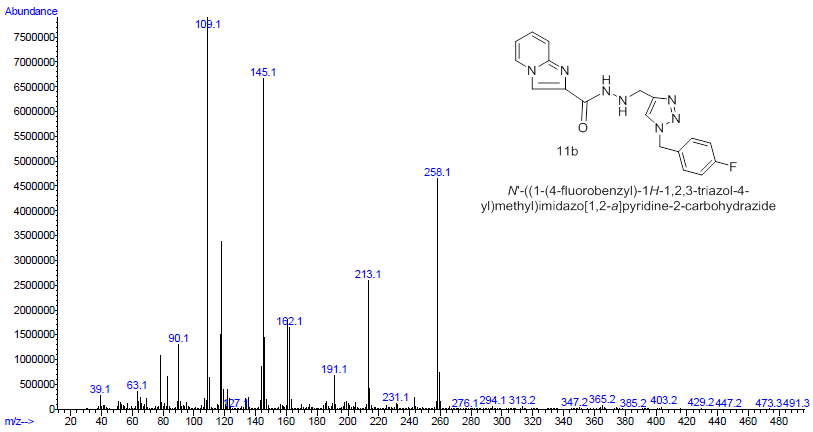
**

Figure S33. MS data of (11b)

**Chemical structure of major Fragment of 11b**


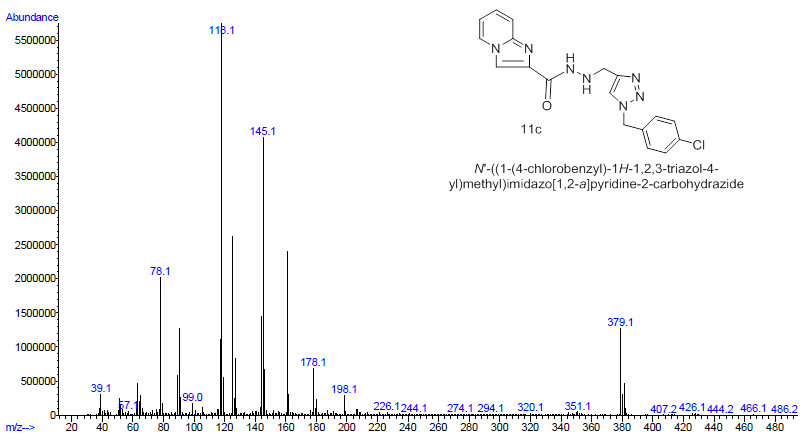


Figure S34. MS data of (11c)


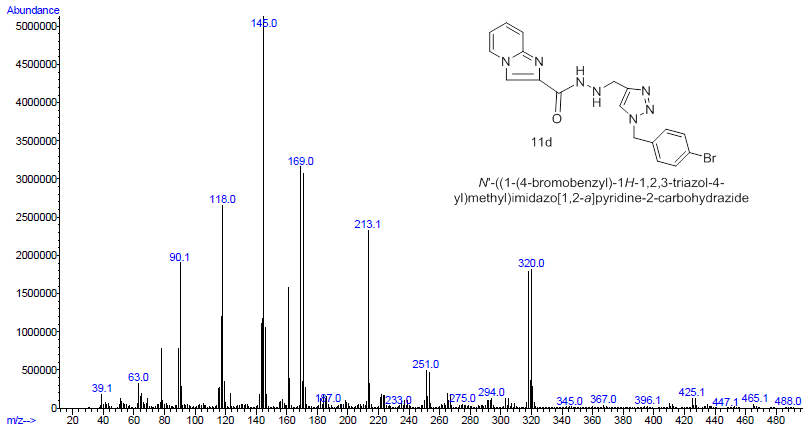


Figure S35. MS data of (11d)


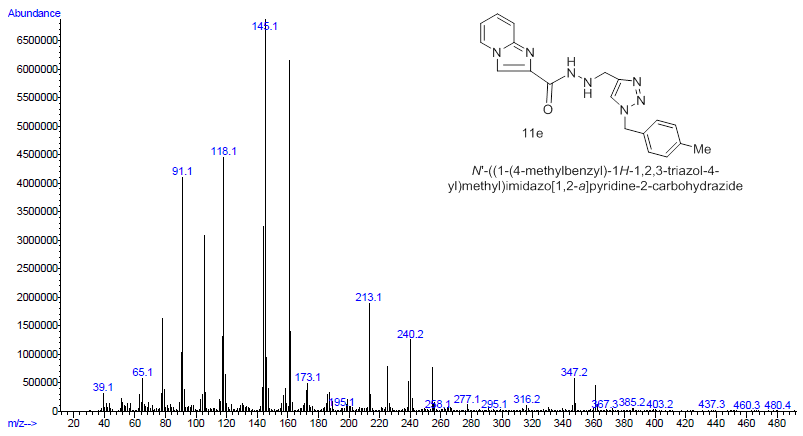


Figure S36. MS data of (11e)
